# A Randomized Controlled Trial of Folate Supplementation When Treating Malaria in Pregnancy with Sulfadoxine-Pyrimethamine

**DOI:** 10.1371/journal.pctr.0010028

**Published:** 2006-10-20

**Authors:** Peter Ouma, Monica E Parise, Mary J Hamel, Feiko O. ter Kuile, Kephas Otieno, John G Ayisi, Piet A Kager, Richard W Steketee, Laurence Slutsker, Anna M van Eijk

**Affiliations:** 1 Centre for Vector Biology and Control Research, Kenya Medical Research Institute, Kisumu, Kenya; 2 Division of Parasitic Diseases, National Center for Infectious Diseases, Centers for Disease Control and Prevention, Atlanta, Georgia, United States of America; 3 Kenya Field Station, Centers for Disease Control and Prevention, Kisumu, Kenya; 4 Liverpool School of Tropical Medicine, Pembroke Place, Liverpool, United Kingdom; 5 Academic Medical Centre, University of Amsterdam, Amsterdam, Netherlands; 6 Malaria Control and Evaluation Partnership in Africa, Program for Appropriate Technology in Health, Batiment Avant Centre, Ferney-Voltaire, France

## Abstract

**Objectives:**

Sulfadoxine-pyrimethamine (SP) is an antimalarial drug that acts on the folate metabolism of the malaria parasite. We investigated whether folate (FA) supplementation in a high or a low dose affects the efficacy of SP for the treatment of uncomplicated malaria in pregnant women.

**Design:**

This was a randomized, placebo-controlled, double-blind trial.

**Setting:**

The trial was carried out at three hospitals in western Kenya.

**Participants:**

The participants were 488 pregnant women presenting at their first antenatal visit with uncomplicated malaria parasitaemia (density of ≥ 500 parasites/μl), a haemoglobin level higher than 7 g/dl, a gestational age between 17 and 34 weeks, and no history of antimalarial or FA use, or sulfa allergy. A total of 415 women completed the study.

**Interventions:**

All participants received SP and iron supplementation. They were randomized to the following arms: FA 5 mg, FA 0.4 mg, or FA placebo. After 14 days, all participants continued with FA 5 mg daily as per national guidelines. Participants were followed at days 2, 3, 7, 14, 21, and 28 or until treatment failure.

**Outcome Measures:**

The outcomes were SP failure rate and change in haemoglobin at day 14.

**Results:**

The proportion of treatment failure at day 14 was 13.9% (19/137) in the placebo group, 14.5% (20/138) in the FA 0.4 mg arm (adjusted hazard ratio [AHR], 1.07; 98.7% confidence interval [CI], 0.48 to 2.37; *p* = 0.8), and 27.1% (38/140) in the FA 5 mg arm (AHR, 2.19; 98.7% CI, 1.09 to 4.40; *p* = 0.005). The haemoglobin levels at day 14 were not different relative to placebo (mean difference for FA 5 mg, 0.17 g/dl; 98.7% CI, −0.19 to 0.52; and for FA 0.4 mg, 0.14 g/dl; 98.7% CI, −0.21 to 0.49).

**Conclusions:**

Concomitant use of 5 mg FA supplementation compromises the efficacy of SP for the treatment of uncomplicated malaria in pregnant women. Countries that use SP for treatment or prevention of malaria in pregnancy need to evaluate their antenatal policy on timing or dose of FA supplementation.

## Editorial Commentary

**Background:** Health authorities worldwide recommend that pregnant women supplement their diet with folate (one of the B-vitamins), normally 0.4 mg per day. There is good evidence from systematic reviews of controlled trials that folate supplementation around conception and early in pregnancy is effective in protecting against neural tube (spine and brain) defects; continued supplementation throughout pregnancy reduces the chance of anemia in the mother. In many African countries, including Kenya, the dose of folate used is 5 mg per day, because this dose is more easily available there. In Kenya, as well as elsewhere in Africa, sulfadoxine-pyrimethamine is also given twice or more after the first trimester to treat and/or prevent malaria infection (which is more likely, and can have serious consequences, when a woman is pregnant). However, there is some evidence from laboratory experiments and clinical studies, none of which were done in pregnant women, suggesting that folate supplementation might reduce the effectiveness of sulfadoxine-pyrimethamine. Therefore, these researchers conducted a trial to test this hypothesis in 415 pregnant Kenyan women with malaria parasites in the blood but no severe symptoms. All were given standard sulfadoxine-pyrimethamine treatment. The women were randomized to receive either folate 5 mg daily, folate 0.4 mg daily, or placebo tablets for 14 days, after which all women reverted to the standard folate 5 mg tablets. The women were followed up for 28 days after the initial sulfadoxine-pyrimethamine dose and the principal outcome the researchers were interested in was the failure of sulfadoxine-pyrimethamine treatment, defined as fever and the presence of parasites in the blood (clinical failure) or the failure of parasites to clear from the blood or to reappear too soon (parasitological failure).

**What this trial shows:** In this trial, women receiving folate 5 mg daily were approximately twice as likely to fail treatment with sulfadoxine-pyrimethamine than women receiving folate 0.4 mg or placebo. (Overall, around 27% of the women receiving folate 5 mg had treatment failure during the follow-up period.) All the treatment groups had similar levels of blood hemoglobin at the end of the study. There did not seem to be any major differences in adverse events (such as premature deliveries, stillbirths, or neonatal deaths) among women taking part in the different study groups.

**Strengths and limitations:** The randomization procedures were appropriate and procedures were used to blind participants and researchers to the different interventions, therefore reducing the risk of bias. Since the trial had a placebo arm, it was possible to conclude that the lower dose of folate (0.4 mg) did not significantly affect efficacy of sulfadoxine-pyrimethamine as compared with placebo. A limitation of the study is that the length of the intervention was short, since all women reverted to standard 5 mg folate after 14 days. It is therefore not clear whether a longer trial would have shown additional risks or benefits of the different doses of folate. Finally, PCR genotyping was not done on the parasites infecting women in the trial; this procedure could have distinguished between true treatment failures and new infections (but which would have been unlikely within 14 days).

**Contribution to the evidence:** Other trials and observational studies have suggested that high doses of folate can reduce the efficacy of sulfadoxine-pyrimethamine in children and adults. However these studies have not examined the effect in pregnant women, for whom most national bodies recommend regular folate supplementation. The results from this trial supports the findings from previous studies and enables the evidence to be generalized to pregnant women. The study also found no evidence that 0.4 mg folate compromises the efficacy of sulfadoxine-pyrimethamine. The findings suggest that the lower level of folate dosing should be used in pregnancy, or that antimalarial treatments other than sulfadoxine-pyrimethamine be used.

## INTRODUCTION

In malaria endemic areas in sub-Saharan Africa, pregnant women are more likely to be infected with Plasmodium falciparum than nonpregnant women, affecting approximately 30 million pregnancies annually [1,2]. Adverse consequences of malaria in pregnancy include maternal anaemia, maternal mortality, low birth weight of the infant, and foetal loss [3,4]. The World Health Organization recommends three interventions for the control of malaria in pregnancy in areas of stable transmission: intermittent preventive treatment, the use of insecticide treated nets, and case management of malarial illness and anaemia [5]. Many countries in sub-Saharan Africa use sulfadoxine-pyrimethamine (SP) for the treatment of clinical malaria in pregnancy or have introduced intermittent preventive treatment in pregnancy (IPTp) with SP as national policy [5]. IPTp consists of two or more presumptive treatment doses of SP after the first trimester delivered through the antenatal clinic, and has been shown to reduce adverse effects of malaria in pregnancy [6–11]. Kenya adopted this policy in 1998.

Folate (FA) supplementation in pregnancy has been associated with reduction in anaemia and prevention of megaloblastic erythropoiesis [12]; it is universally recommended as part of antenatal care. Although international guidelines recommend 0.4 or 0.6 mg of FA daily [13–15], many countries in sub-Saharan Africa, including Kenya, use 5 mg FA daily [16], because the 5 mg tablet is more widely available.

In areas of malaria transmission, IPTp with SP and FA are often coadministered as part of antenatal care. However, the mode of action of SP is based on the competitive inhibition of two key enzymes in the biosynthesis of FA by the malaria parasite. Several studies have shown that FA can antagonize the antimalarial activity of SP in vitro and in vivo [17–21]. These studies, although not conducted among pregnant women, have resulted in some public health authorities recommending that FA should be temporarily withheld after SP administration. However, temporary suspension of folate makes program implementation complicated and may not be necessary.

We conducted a randomized, double-blind, placebo-controlled study among pregnant women with uncomplicated malaria to assess whether FA 5 mg compromises the efficacy of SP, and if a low dose of FA, such as 0.4 mg, may be an acceptable alternative. The effect of maternal HIV infection will be discussed in a separate manuscript.

## METHODS

### Participants

This study was conducted at three government hospitals in western Kenya: Nyanza Provincial General Hospital in the Kisumu District (population 500,000); Bondo District Hospital (district population 300,000), and Siaya District Hospital (district population 480,000). In each site, HIV counselling and testing is provided in the antenatal clinic as part of a program to provide nevirapine to HIV-seropositive pregnant women to reduce vertical transmission of HIV. Malaria transmission is perennial and intense in western Kenya; however, the malaria prevalence among pregnant women in Kisumu is lower than in the rural areas of Bondo and Siaya. Participants were recruited from the daily antenatal clinics in the participating hospitals; the inclusion and exclusion criteria are summarized in Table 1. The study protocol was approved and reviewed on an annual basis by the institutional review boards of the Kenya Medical Research Institute, and the Centers for Disease Control and Prevention, Atlanta, United States. All participants gave informed consent.

**Table 1:**
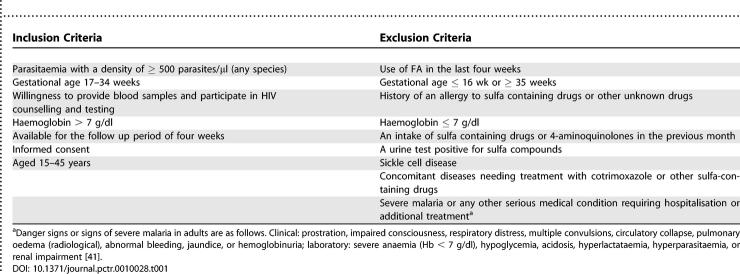
Inclusion and Exclusion Criteria

### Interventions

A study nurse or clinical officer randomized participants to FA 5 mg tablets (FA 5 mg arm), FA 0.4 mg tablets (FA 0.4 mg arm), or placebo tablets (FA placebo arm); all were identical in appearance and taste (Laboratory and Allied, Nairobi, Kenya). Participants received a 14-day supply. At day 14, all women received a supply of folic acid 5 mg tablets for 14 days to ensure that pregnant women were not deprived of FA. The first doses of FA or placebo were given together with SP (three tablets of Malodar [Laboratory and Allied]: 1,500 mg of sulfadoxine and 75 mg of pyrimethamine at once) under supervision. Participants were observed for half an hour; if vomiting occurred, the SP dose and FA tablet were repeated. Participants were instructed to take the FA or placebo tablet daily and were asked to bring the tablets at every visit for a tablet count. All participants were supplemented with iron tablets according to the national guidelines (200 mg three times per day). From August 2004 onwards, all participants received insecticide-treated nets (ITNs) as part of the enrolment procedure to reduce the chance of new malaria infections. Participants were instructed to return to the clinic on days 2, 3, 7, 14, 21, and 28, or whenever they felt ill and thought they needed treatment. On follow-up visits, women were questioned about side effects, and signs and symptoms of clinical malaria. The axillary temperature was measured, and blood was obtained for a malaria blood smear; haemoglobin was repeated on days 14 and 28. Women who were ill or had complications that did not allow them to continue participation were referred to the appropriate departments in the hospital and followed until recovery. Women who failed treatment with SP received quinine 600 mg three times per day for seven days. Women who had not cleared parasitaemia after seven days of quinine therapy were treated with mefloquine.

Haemoglobin was measured to the nearest 0.1 g/dl using a portable haemoglobin monitor (HaemoCue, Mission Viejo, California, United States). Peripheral thick and thin blood films were stained with 10% Giemsa, and examined under oil immersion for malaria parasites. A thick film was considered negative if 100 microscopic fields showed no parasites. Malaria parasites and leukocytes were counted in the same fields until 300 leukocytes were counted. Parasite densities were estimated by assuming a count of 8,000 leukocytes/μl of blood. For quality control of the blood smear reading, 10% of the negative samples and 20% of the positive samples at screening, and 20% of all follow-up samples were checked by a different microscopist during the study. HIV testing involved parallel use of two rapid testing methods: Determine HIV-1/2 (Abbott Laboratories, Dainabot, Tokyo, Japan) and Unigold HIV-1/2 (Trinity Biotech, Bray, Ireland), as per Kenya Ministry of Health guidelines for voluntary counselling and testing. Capillus HIV-1/2 (Cambridge Diagnostics, Wicklow, Ireland) was performed on discordant samples. The method of Mount et al. [22] was used to test the urine for sulfa compounds. The sickle cell profile was determined using cellulose acetate electrophoresis (Helena Laboratories, Beaumont, Texas, United States).

### Objectives

We investigated whether FA supplementation in a high or a low dose affects the efficacy of SP for the treatment of uncomplicated malaria in pregnant women.

### Outcomes

Outcome measures were the prevalence of SP treatment failure at days 3, 7, 14 (primary outcome), and 28 and change in haemoglobin level comparing day 0 (day of SP treatment) to days 14 and 28. Treatment failures were defined according to the guidelines for an area of low to moderate transmission (Table 2) [23]. The main difference from the protocol for areas of high transmission is that in the moderate transmission protocol an afebrile patient who still had parasitaemia on day 7 post-treatment was classified as a late parasitological failure and given rescue treatment, whereas such patients would not have been classified as parasitological failures in the high-transmission protocol. Because of the adverse consequences that asymptomatic parasitaemia can have for mother and foetus, we decided to use the more conservative protocol.

**Table 2:**
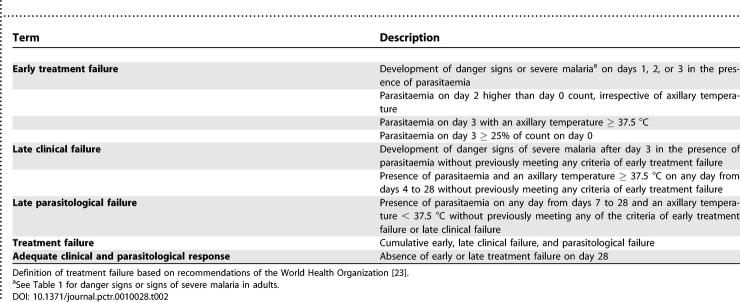
Definition of Treatment Failure

Malaria was defined as the presence of asexual-stage parasite of any species in thick smears, independent of clinical signs. A parasite density in the highest tercile at enrolment of the total study population was defined as a high parasite density. A young age was defined as under 20 years. Because of the short duration of follow-up and limited sample size, no attempt was made to assess the effect of FA on megaloblastic anaemia.

### Sample Size

We calculated that a sample size of 600 women—200 in each arm—would allow us to detect an increase from 5% to 15% in the parasitological failure rate at day 7 with 80% power and 95% confidence, allowing for a 25% loss to follow-up. However, we did not feel comfortable continuing the trial at an overall treatment failure rate of over 40% at day 28 and an intervention which may contribute to this, because it is recommended that first-line therapy be changed at a 25% failure rate [23,24]. An interim analysis was performed in October 2005 with stopping criteria defined as a difference in the treatment failure rate at day 14 with a *p*-value of less than 0.01; we used day 14 and not day 7 because we considered day 14 a more appropriate time point for assessing SP resistance. Because this criterion was met, enrolment was stopped with 488 women enrolled. The investigators remained blind until data cleaning, analysis, and quality control of the blood smears were completed.

### Randomization—Sequence Generation

One of the investigators generated a randomization list with a block size of 12 using the statistical program SAS (SAS system for Windows version 8; SAS, Cary, North Carolina, United States).

### Randomization—Allocation Concealment

All FA treatment and placebo tablets were prepared off site and were identical in appearance and taste (Laboratory and Allied). Medicine envelopes with 14 tablets of a treatment arm were prepacked and labelled with the arm by staff who were not involved in randomization. The medicine envelopes were put in sealed, opaque envelopes with consecutive numbers according to the randomization list by an investigator.

### Randomization—Implementation

A trained clinical officer or nurse randomized eligible women by assigning them the next envelope in order of enrolment. The envelope was opened by the participant, and the study arm was allocated by the study staff according to the arm indicated on the medicine envelope.

### Blinding

All FA treatment and protocol tablets were prepared off site and were identical in appearance and taste (Laboratory and Allied). All study staff participants were blind to the treatment in each arm.

### Statistical Methods

We analysed the data on an intention-to-treat basis using a pre-established analysis plan. Cumulative treatment failures by follow-up day were compared among treatment arms using the Chi-squared test. We used the Kaplan-Meier curve to examine differences in patterns between treatment arms, and Cox proportional hazards regression analysis to examine the effect of treatment arm on time to treatment failure after we confirmed that the Cox proportional hazard assumption was met. For day 14 post-treatment, we repeated the Cox proportional hazards regression while adjusting for potential confounders; factors examined included site of enrolment, the use of an ITN, ethnicity, education level, socioeconomic status, sickle cell status (carrier versus not a carrier), gravidity, young age, HIV status, and a high parasite density at enrolment. Analysis of covariance was used to assess the effect of treatment arm on haemoglobin level [25]. Factors were removed from models if the *p*-value was 0.05 or more. The statistical program SAS (SAS system for Windows version 8) was used for all analyses. All tests were two-sided; *p* < 0.05 was considered significant, except for the efficacy between study arms when a *p* < 0.013 was considered significant to adjust for multiple comparisons and the interim analysis (the *p*-value of 0.05 was subtracted by 0.01 to account for the interim analysis, and the remaining value was divided by 3 to account for the comparisons between the arms); a confidence interval (CI) of 98.7% was used for the efficacy analysis.

## RESULTS

### Participant Flow

**Figure 1:**
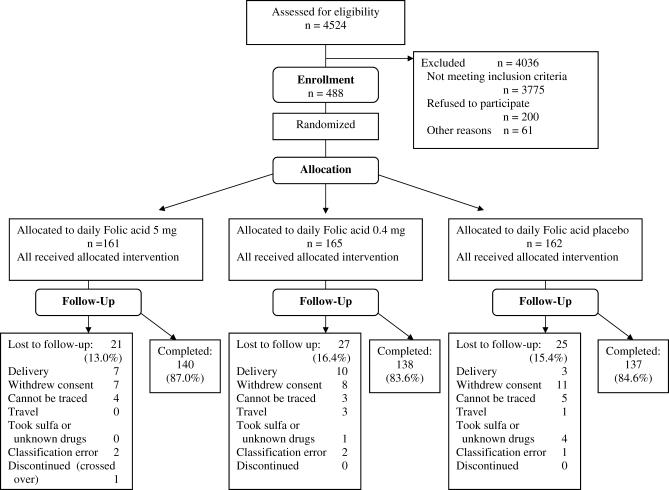
Trial Profile of the Study

Between November 2003 and November 2005, a total of 4,524 women were screened; 488 met all enrolment criteria, and 415 (85%) women completed the study (Figure 1). The study arms were similar in baseline characteristics (Table 3). Most infections were Plasmodium falciparum (98.0%), nine were mixed P. falciparum/*P. malariae,* and one was pure P. malariae. During 1,671 (99.4%) of the 1,682 routine visits made at or before day 14, the participant reported that she took the FA daily; the tablets were brought at 1,454 of the routine visits (86.4%) and a correct count was established at 1,307 visits (89.9%).

**Table 3:**
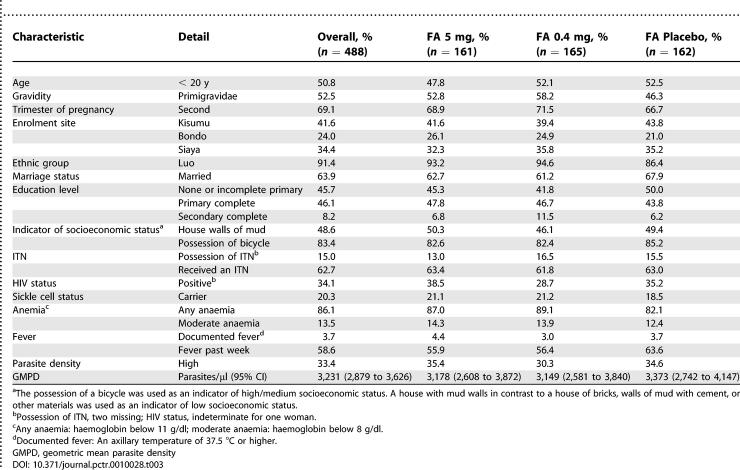
Characteristics of Study Population at Enrolment, Overall and by Treatment Arm

### Outcomes and Estimation

**Figure 2:**
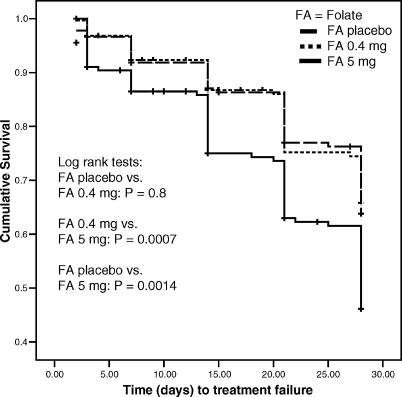
Cumulative Treatment Survival Rates by Intervention Arm among Parasitaemic Pregnant Women Treated with SP and FA Participants received the FA intervention up to 14 days past SP treatment; after day 14 every participant received FA 5 mg in accordance with the National Guidelines in Kenya.

From day 3 onwards, women in the FA 5 mg arm were more likely to fail treatment than women in the other arms (Figure 2; log rank test *p* < 0.01 comparing the FA 0.4 mg arm or the FA placebo arm to the FA 5 mg arm). On day 14 the number of treatment failure was 38 out of 140 women (27.1%) in the FA 5 mg arm, 20 out of 138 women (14.5%) in the FA 0.4 mg arm, and 19 out of 137 women (13.9%) in the FA placebo arm (Table 4). In multivariate analysis using Cox proportional hazards regression, compared to FA placebo, treatment failure by day 14 was twice as likely when FA 5 mg was used (hazard ratio [HR], 2.19; 98.7% CI, 1.09 to 4.40; *p* = 0.005), whereas FA 0.4 mg did not affect treatment failure risk (HR, 1.07; 98.7% CI 0.48 to 2.37; *p* = 0.8) (Table 5).

**Table 4:**
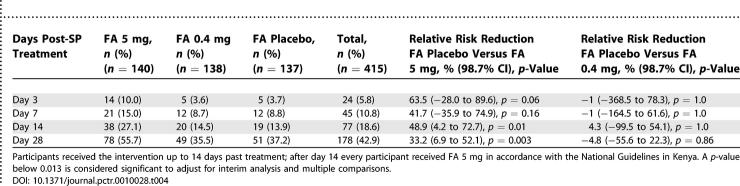
Cumulative Treatment Failures and Relative Risk Reduction by Follow-Up Day among Participants Who Completed the Study

**Table 5:**
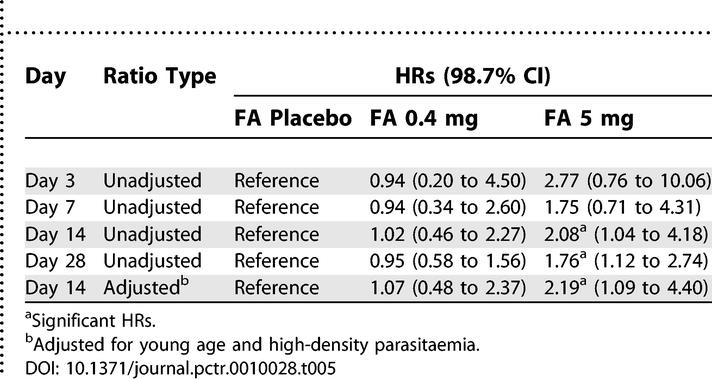
The Effect of Daily FA Dose on Time to Treatment Failure of SP among Pregnant Women by Follow-Up Time Point

**Figure 3:**
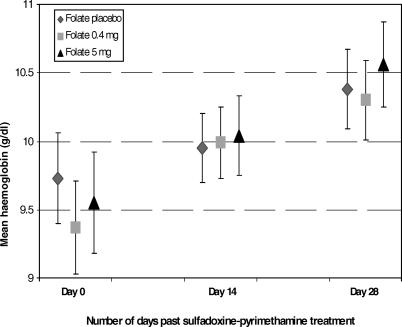
Haemoglobin Levels by Intervention Arm at Different SP Treatment Time Points Haemoglobin levels (mean and 98.7% CI) are shown by type of FA intervention at enrolment, 14 days, and 28 days post-treatment with SP for malaria among 287 pregnant women who completed 28 days of follow-up without treatment failure. Mean haemoglobin was obtained by analysis of covariance and was adjusted for HIV, site of residence (rural versus urban), and high parasite density. On days 14 and 28 the haemoglobin was adjusted for haemoglobin at enrolment as well. Participants received the intervention up to 14 days past SP treatment; after day 14 every participant received FA 5 mg in accordance with the National Guidelines in Kenya

We did not find an effect of treatment arm on haemoglobin levels at day 14 or day 28 among 288 women who completed 28 days of follow-up without treatment failure (Figure 3). Among 386 women who had a haemoglobin available at day 14, the increases in mean haemoglobin in the FA 5 mg and FA 0.4 mg arms were not statistically different compared to the FA placebo arm (0.17 g/dl; 98.7% CI, −0.19 to 0.52 g/dl; *p* = 0.3, and 0.14 g/dl; 98.7% CI, −0.21 to 0.49 g/dl; *p* = 0.4, respectively; adjusted for maternal HIV infection, location of residence, high parasite density infection, and haemoglobin at enrolment).

### Adverse Events

During the course of the study, 20 participants (4.1%) developed rashes (4, 8, and 8 in the FA 5 mg, FA 0.4 mg, and FA placebo arms, respectively). No severe adverse skin reactions or maternal deaths occurred. Premature delivery was experienced by 14 participants (2.9%) (6, 5, and 3 in the FA 5 mg, FA 0.4 mg, and FA placebo arms, respectively), and eight participants (1.6%) had a stillbirth or early neonatal infant death during the study (3, 2, and 3 in the FA 5 mg, FA 0.4 mg, and FA placebo arms, respectively).

## DISCUSSION

### Interpretation

This study shows that the combined use of SP and daily FA supplementation in a dose of 5 mg compromised the efficacy of SP for the treatment of malaria parasitaemia in pregnant women. A plausible biological mechanism is available. FA is required for DNA synthesis in both humans and protozoa. Malaria parasites can utilize exogenous FA (the salvage pathway) as well as synthesize FA de novo (biosynthesis) [26], though biosynthesis seems to be the preferred method [27]. Antifolates such as SP act on two enzymes important for sequential steps in the biosynthesis of FA for the parasite, dihydropteroate synthase and dihydrofolate reductase, respectively. It has been established that malaria parasites can differ in their ability to use exogenous FA, but the mechanism is unknown [28,29]. If the biosynthesis pathway is compromised, e.g., by sulfadoxine, parasite strains that are able to use exogenous FA can compensate for the lack of FA through the biosynthesis pathway by increasing the flux through the FA salvage pathway [27,28]. However, pyrimethamine may interfere with the utilization of exogenous FA in a competitive way, an action that is thought to be independent of pyrimethamine's inhibition of dihydrofolate reductase [28,30]. The success and duration of the effect of pyrimethamine may be dependent on the FA levels; large amounts of FA (such as 5 mg daily), but not low doses, may overwhelm pyrimethamine's ability to block the salvage pathway [30].

It is likely that FA supplementation affects other antifolate antimalarial combinations as well, such as chlorproguanil-dapsone, dapsone-pyrimethamine, and cotrimoxazole. Cotrimoxazole will increasingly be used as a prophylactic drug among HIV-positive pregnant women. Further study into the effect of concomitant FA supplementation in malarious areas is needed.

We did not collect blood at enrolment and follow-up visits to be able to differentiate between recrudescent and new malaria infections. After day 14, all groups switched to FA 5 mg, so we were not able to assess the extent to which FA contributes to SP treatment failure after 14 days. Several studies indicate that FA supplements do not predispose to increased risk of malaria acquisition [31,32], and thus we hypothesized that the difference between treatment arms as observed in this study after day 14 is mainly caused by recrudescence.

### Overall Evidence

Our results are supported by studies among symptomatic, nonpregnant persons in areas of different malaria endemicity. A randomized, placebo-controlled study in Gambia reported approximately twice as common SP treatment failures among children with symptomatic malaria supplemented with a high dose of FA (5 mg daily for children < 15 kg, 7.5 mg daily for children 15–20 kg, and 10 mg daily for children > 20 kg) compared to the FA placebo group in an area with low seasonal malaria transmission [19]. In a low-to-moderate malaria transmission area in Kenya, a randomized, open-label study among symptomatic participants (all ages) showed a comparable cumulative survival curve when assessing the interaction of SP and FA (5 mg daily) [20]. Dzinjalamala et al. [21] recently noted significantly higher mean FA levels at enrolment among children with a treatment failure to SP for symptomatic malaria (28 day follow-up) compared to children with an adequate parasitological and clinical response in Malawi.

### Generalizability

SP is recommended for the treatment and prevention of malaria in pregnancy. Although our results are based on the treatment of uncomplicated malaria in pregnant women, they will have implications for the use of SP as IPTp as well. Many countries have introduced IPTp with SP [5]. We cannot assess from our study the effect of FA 5 mg on the preventive action of SP on malaria, but FA 5 mg will affect the treatment action of SP when malaria parasitaemia is present. Depending on the endemicity of malaria in an area, it can be expected that 1%–50% of pregnant women may carry malaria parasitaemia, without noticing it, particularly in the placenta [1,3]. Given the present results, countries using IPTp should consider evaluating their FA recommendations in the antenatal clinic to optimize SP efficacy. Options to consider include using low-dose (0.4 mg) FA tablets daily or suspending FA 5 mg for 14 days after SP treatment, which would disrupt an important routine of daily intake of FA for the prevention of anaemia. The first option may be preferable; our data show no difference in efficacy of SP between 0.4 mg FA daily and withholding FA 5 mg for 14 days. Effects of regimens on haemoglobin levels were similar. However, this study was not designed to assess the optimal FA dose to prevent FA deficiency and adverse events such as megaloblastic anaemia in the presence of malaria parasitaemia. International guidelines recommend folic acid doses of 0.4 or 0.6 mg daily during pregnancy [13–15]. Although these international recommendations for FA supplementation are based on studies conducted in developed countries, the few studies in sub-Saharan Africa assessing FA deficiency among pregnant women suggest that such deficiency is relatively uncommon, ranging from 3%–10%; an exception was Togo (68% FA deficiency among pregnant women) [33–38]. A dose of 1 mg of FA daily in combination with malaria prophylaxis was sufficient to abolish FA deficiency among primigravidae in Zaria, Nigeria [39]. Given the international recommendations, the relatively low prevalence of FA deficiency in pregnancy, and the compromised efficacy of SP for malaria treatment when FA 5 mg is used, we believe it is reasonable to recommend FA 0.4 mg daily for pregnant women in malarious areas in sub-Saharan Africa.

Resistance to SP was high in the study area. However, at present, no safe and efficacious alternative drug is available for the treatment and prevention of malaria in pregnancy. Kenya has moved now to artemisinin-based combination therapy for children and quinine as first-line therapy for clinical malaria in pregnancy, but IPTp with SP continues to be used for prevention. A recent review of the efficacy of IPTp with SP in the face of increasing SP resistance reported that in areas with parasitological failure as high as 30% at day 14 in children under 5 years of age, significant reductions in adverse effects of malaria in pregnancy were seen when IPTp was used [6–8]. However, alternatives for IPTp with SP urgently need to be investigated. Considering the unique properties of SP in its combination of treatment and prevention, including its low cost and affordability, it may be worthwhile to preserve SP for use as IPT among pregnant women or infants in areas where SP resistance is low, and to use combination therapy with other antimalarials for the treatment of symptomatic malaria, reducing the drug pressure in the community treatment rate [40]. Ensuring that the action of SP is not compromised by concurrent high-dose FA supplementation will further increase its therapeutic life.

## SUPPORTING INFORMATION

CONSORT Checklist Click here for additional data file. (50 KB DOC)

Trial Protocol Click here for additional data file. (298 KB DOC)

## Abbreviations

AHR: adjusted hazard ratio
CI: confidence interval
FA: folic acid
HR: hazard ratio
IPTp: intermittent preventive treatment in pregnancy
ITN: insecticide-treated net
SP: sulfadoxine-pyrimethamine
