# Supplementary material for: A Randomized Controlled Trial of Folate Supplementation When Treating Malaria in Pregnancy with Sulfadoxine-Pyrimethamine
Source: PLoS Clin Trials. 2006 Oct 20;1(6):e28. doi: 10.1371/journal.pctr.0010028 (PMC1617124; doi:10.1371/journal.pctr.0010028)
Supplement: Trial Protocol [file pctr.0010028.sd002.doc]

**I. Title**

THE EFFECT OF FOLIC ACID SUPPLEMENTATION ON EFFICACY OF SULFADOXINE-PYRIMETHAMINE IN PREGNANT WOMEN IN WESTERN KENYA.

**II. Investigators & Institutions**

**A Principal Investigators**

A. M. van Eijk^1, 2^

M. E. Parise^2^

L. Slutsker^1, 2^

**B Investigators**

J. G. Ayisi^1^

D. H. Rosen^1,2^

F. O. ter Kuile^2^

Y. Shi^1,2^

A. O. Misore^3^

J. Odondi^3^

J. A. Otieno^3^

R. W. Steketee ^2^

M. Hamel^1,2^

P. Ouma^1,2^

1. Kenya Medical Research Institute (KEMRI), Vector Biology and Control Research Center (VBRC), Kisumu, Kenya

2. Division of Parasitic Diseases, National Center for Infectious Diseases (NCID), Centers for Disease Control and Prevention (CDC), Atlanta, USA

3. Kenya Ministry of Health, Kisumu, Kenya

**III. Summary**

In malaria endemic areas in sub-Saharan Africa, pregnant women, especially primi - and secundigravidae, are more likely to have placental and peripheral parasitemia with *Plasmodium falciparum* than non-pregnant women. Adverse consequences of malaria in pregnancy include maternal anemia, and low birth weight of the new born. Low birth weight is known to be the most important risk factor for infant mortality.

Due to widespread and high-grade chloroquine resistance, sulfadoxine-pyrimethamine (SP) is rapidly replacing chloroquine in East Africa as first line drug for treatment of malaria. Recent studies have shown that intermittent preventive treatment (IPT) with SP during pregnancy can mitigate the adverse effects of malaria in pregnancy. In 1998 The Kenya National Malaria Control Program adopted the strategy of IPT with SP to be delivered as one treatment dose of SP at the first antenatal clinic visit occurring after the first trimester, and a second treatment dose of SP at the beginning of the third trimester (between 28 and 34 weeks).

The action of SP is based on inhibition of parasite enzymes in the metabolism of folic acid. However, in vitro studies indicate that folic acid can antagonize the antimalarial parasite activity of SP. Furthermore, in one recent West African study, supplementary folic acid compromised the antimalarial efficacy of SP in children with acute malaria aged 6 months to 12 years.

Folic acid requirements are increased during pregnancy, and supplementation with folic acid in pregnancy is recommended. Although in most countries a daily supplementation of 400 to 600 micrograms is considered sufficient, for logistical reasons the daily recommended dose in Kenya is 5 mg of folic acid during pregnancy. It is unknown whether folic acid supplementation might compromise the efficacy of IPT with SP in pregnant women living in malaria endemic areas.

We propose a placebo-controlled study to address this question. Parasitemic pregnant women visiting the ANC of Nyanza Provincial General Hospital, Bondo District Hospital, or Siaya District Hospital will be randomized to receive either SP with folic acid 5 mg, or SP with folic acid 400 g, or SP and placebo. The placebo and the folic acid 400 g will be given for two weeks, and then be replaced by folic acid 5 mg. Data from this study will be useful to evaluate the need for a modified regimen of folic acid supplementation recommendation in areas where IPT with SP is used.

**IV. Introduction and background**

**Introduction**

Malaria in pregnancy is a problem involving more than 20 million women of childbearing age per year in sub-Saharan Africa (United Nations Children’s Fund, 1998). Compared to non-pregnant women, pregnant women more frequently harbor *Plasmodium falciparum* in the peripheral and placental vascular space, and experience higher density infections. Although in malaria endemic areas severe clinical malaria in pregnancy is not common, this increased frequency and density of *P. falciparum* infection is associated with lower maternal hemoglobin, fetal growth retardation and prematurity, particularly in primigravidae (Brabin 1983, Menendez 1995, Steketee et al, 1996a). Low birth weight is known to be the most important risk factor for infant mortality (McCormick, 1985). Recent estimates suggest that among pregnant women in malaria endemic areas, 3-15% of anemia, 8-14% of low birth weight and 3-8% of infant mortality could be attributed to malaria (Steketee et al, 2001).

Approaches to reduce the impact of malaria in pregnancy have included weekly chemoprophylaxis with chloroquine; however, the feasibility of such a program was compromised by logistical constraints and the bitter taste of the drug (Kaseje et al, 1987). With increasing resistance of *P*. *falciparum* to chloroquine, new approaches were evaluated.

In studies in sub-Saharan Africa, intermittent preventive treatment (IPT) with sulfadoxine-pyrimethamine (SP), one dose in the second trimester and one dose in the early third trimester (28-34 weeks), was found to reduce the prevalence of parasitemia (Schultz et al, 1994, Parise et al, 1998), maternal anemia (Shulman et al, 1999) and low birth weight (Parise et al, 1998, Verhoeff et al, 1998, Rogerson et al, 2000). Studies that reported information on side effects documented that IPT was well tolerated; side effects, if they occurred, were mild and no severe adverse skin reactions were seen. The use of SP was not associated with neonatal complications, like kernicterus in the newborn, or congenital abnormalities (Verhoeff et al, 1998, Parise et al, 1998).

Currently, the World Health Organization recommends that pregnant women in malaria endemic areas receive IPT: the administration of full, curative-treatment doses of an effective antimalarial drug at predefined intervals during pregnancy (WHO 2000). In areas such as sub-Saharan Africa with substantial chloroquine resistance, interest has focused on the delivery of SP as IPT. Besides the relatively low costs of a treatment dose in comparison with other antimalarials, SP has the advantage of being a single dose drug; the three tablets (adult dose) can be given under supervision in the antenatal clinic (ANC), leading to improved adherence over regimens requiring dosing over multiple days.

In 1993, Malawi was the first country to introduce IPT with SP for the control of malaria in pregnancy as a national policy. In 1998, Kenya adopted this policy, and more African countries are adopting or considering this approach.

**Folic acid and pregnancy**

Human beings are fully dependent on dietary sources or dietary supplements for their folate supply. Folate is an essential nutrient for cell metabolism and for the synthesis of purines and pyrimidines, components of DNA and RNA. During pregnancy, folate is necessary for normal embryonic development and growth. Recent research has demonstrated a significant increase in folate catabolism during pregnancy, with a peak in the third trimester and a sharp fall after delivery, corresponding with the period of maximal increase in fetal mass (Higgins et al, 2000). Folic acid deficiency in pregnancy has been associated with the development of fetal neural tube defects, maternal megaloblastic anemia, and low birth weight (Lumley et al, 2001, Smits & Essed 2001, Mahomed 2000). Recommendations for doses of folic acid to be taken during pregnancy differ per country and purpose.

Supplementation with folic acid around the time of conception significantly reduced the incidence of neural tube defects but folate supplementation begun after the first trimester of pregnancy is too late to prevent birth defects (Lumley et al, 2001). In the USA, the recommended dose of folic acid to prevent neural tube defects is 400 microgram per day, starting preferably one month before conception and continuing throughout the first three months of pregnancy (CDC 1992). However, a recent review recommends even 5 mg folic acid tablets daily to reduce the risk of neural tube defects (Wald et al, 2001).

A Cochrane review specifically addressing the effect of folate supplementation in pregnancy on haematological and biochemical parameters and measures of pregnancy outcome (Mohamed, 2000) reports that compared to placebo or no supplementation, folate supplementation was associated with a significant reduction in the proportion of women with low haemoglobin level in late pregnancy and megaloblastic erythropoiesis. Apart from a possible reduction in the incidence of low birthweight, folate supplementation appeared to have no measurable effect on any other substantive measures of pregnancy outcome (Mohamed, 2000).

For the prevention of folate deficiency and megaloblastic anemia in pregnancy, the recommended dietary allowance is 600 microgram per day (Food and Nutrition Board of the Institute of Medicine, USA, German Nutrition Society). International anemia treatment guidelines recommend in areas with a prevalence of anemia in pregnancy of 40%, 400 microgram folic acid in combination with 60 mg iron daily for 6 months in pregnancy, and continuing 3 months postpartum (Stoltzfus & Dreyfuss, 1997). In Kenya 5 mg of folic acid per day is recommended for prevention of anemia, to start at the first ANC and to continue until labor; 5 mg of folate was chosen because it was the lowest dose in a tablet that was available in the country (Ministry of Health, 1994). In the Kenya MOH simplified national guideline on malaria control for community resource persons, treatment of anemia in pregnant women is recommended to be three months of folic acid 5 mg/day, starting two days after any SP (Ministry of Health, 2000). The WHO recommends a delay folic acid supplementation for one week after SP to avoid an inhibitory effect on antimalarial efficacy (WHO expert committee on malaria, 2000).

The evidence available on the role of folate in reducing the risk of vascular disease, cancer and psychiatric and mental disorders is not sufficiently conclusive to be able to come up with recommendations for supplementation (The National Academy of Sciences, 2000).

**Malaria and folic acid**

There is evidence from several studies that red blood cell folate concentrations may be raised in the presence of malaria. First reported in experimental animal systems (Trager, 1959), studies on red blood cell folate concentrations in Nigerian children (Bradley-Moore et al, 1985) and in Kenyan primigravidae (Brabin et al, 1986) confirmed this association. Brabin et al (1986) suggest several explanations. Individuals with malaria who are actively hemolyzing may have an increase in reticulocytes, cells with a much higher concentration of folic acid than mature red blood cells. Other explanations are that parasites may synthesize the folate in the red blood cells, or the host may express an altered metabolism of folic acid in the red blood cell in the presence of malaria. In one in-vitro study, it was suggested that folic acid deficient persons may have some natural resistance to malaria (Brockelman et al, 1983).

If folic acid deficiency were protective against malaria, an association might be seen between malaria parasitemia and the high serum or RBC folate levels achieved with folic acid supplementation. However, this is not supported by results from several other studies. Folate status (serum and red cell folate level) was not significantly related to the density of malaria infection in a cross-sectional study in a representative sample of a rural population in South Benin (Hercberg et al, 1986). Pregnant women in Nigeria who were supplemented with high doses of folic acid (5 injections of 15 mg folic acid each, spread over 2 weeks) did not show a higher parasite prevalence or density after 2 weeks compared to a control group (Gail & Herms 1969). There was no significant difference in parasitemia between Gambian children who received folate supplementation (5 mg/day fortnightly) or placebo (Fuller et al, 1988).

If malaria parasitemia increases red blood cell folate level, treatment of malaria should result in a decrease of the red blood cell folate level, and this was reported after treatment with chloroquine in Kenyan pregnant women (Brabin et al, 1986). However, in a placebo controlled study in Gambian children aged 3 months to 4 years, fortnightly maloprim (dapsone-pyrimethamine) as prophylaxis was associated with a non-significant increase of levels of red cell folate compared to placebo (Fuller et al, 1988). In addition, a study among primigravidae in Nigeria using weekly 5 mg folic acid or placebo showed that malaria treatment and prophylaxis given in the second trimester without folic acid supplementation stabilized folic acid levels compared to pregnant women who did not receive folic acid and antimalarial treatment / prophylaxis (Fleming et al, 1968). In this study no difference in hemoglobin level was seen between folic acid supplemented and non-supplemented pregnant women. The authors postulate that treatment of malaria in pregnancy reduces haemolysis and the requirements for folic acid (Fleming et al, 1968).

**SP and folic acid**

The components of SP, sulfadoxine and pyrimethamine, act at sequential steps of the malaria parasite folate metabolism, resulting in a synergistic inhibition of parasite growth. The action is based on reversible inhibition of enzymes in the metabolism of the malaria parasite for the use of folic acid (Annex 1): sulfadoxine inhibits the plasmodial dihydropteroate synthase (DHPS) and pyrimethamine blocks the dihydrofolate reductase (DHFR). As a result, parasite growth and multiplication in red blood cells is inhibited. Because the enzymes are located in sequence on the plasmodial folate biosynthetic pathway, the selective chemotherapeutic efficacies of the individual components are potentiated. As a consequence, the dose of both components can be reduced and accordingly the risk of side effects decreased. The development of plasmodial resistance against SP may be delayed because the different components affect different enzymes (Weidekamm 1982). An association between stage of parasite development and therapeutic response has been shown for SP, whereby parasite clearance was slower when young ring form of *P. falciparum* predominated at the time of presentation (Rieckmann et al, 1987). For SP to be most effective, the parasite needs to be intracellular, and in the phase of multiplication (schizogony), when purine precursors required for nucleic acid synthesis are formed.

Although sulfadoxine-pyrimethamine is supposed to selectively block parasite enzymes, it is possible that pyrimethamine acts on human DHFR as well, and thus has the potential to induce folic acid deficiency. Pyrimethamine in combination with dapsone has been reported to cause severe megaloblastic anemia when taken in excessive amounts (Hughes & Gatus, 1979). However, mean red cell folate levels were similar in children, aged 3 months to 5 year, who received fortnightly 12.5 mg pyrimethamine and 50 mg dapsone or placebo as prophylaxis for malaria (Fuller et al, 1988). Latent folate deficiency has been reported in expatriates who took one tablet of SP a week for prophylaxis; the authors attribute this however to an effect of sulfadoxine, because decreased mean serum folate levels were not seen in expatriates using pyrimethamine only as prophylaxis (dose of pyrimethamine not reported, Sturchler & Holzer, 1980). For patients who require large doses of pyrimethamine (e.g. for treatment of toxoplasmosis) it is common to advise folinic acid supplementation to reduce this potential effect on folate metabolism. It is not likely that the relatively small amount used for treatment of malaria can induce folic acid deficiency.

Several studies have examined the effect of folic acid on the antimalarial activity of sulfadoxine and/or pyrimethamine. A small clinical study in 9 Gambian children with malaria did not detect an effect of high doses of folic acid over a short period (10 to 30 mg per day for 3 days) on the antimalarial effect of pyrimethamine (0.25 mg/kg body weight, Hurly 1959). Seventy five US marines in Vietnam hospitalized for treatment of malaria received pryimethamine 25 mg 3 times per day for 3 days and sulfisoxazole 500 mg 4 times a day for 6 days, with either folic acid (5 mg per day), folinic acid (5 mg per day), or placebo; there was no difference in parasite clearance among treatment groups (Tong et al, 1970).

Malaria parasites may not be obligatory dependent on p-aminobenzoic acid (PABA) and may also be able to utilize exogenous folate for tetrahydrofolate formation. A study on mechanisms of drug resistance in *P. falciparum* suggested that, once the parasites become resistant to sulfadoxine, they no longer use PABA for dihydrofolate biosynthesis, but that they use folic acid instead (Tan Ariya & Brockelman, 1983, Brockelman et al, 1983). Folic acid antagonized the anti-malaria activity of sulphonamide drugs in vitro (Watkins et al, 1985, Milhous et al, 1985). In the Gambia a higher malaria treatment failure rate was noted among children treated with SP and folic acid compared with children treated with SP and iron, or SP and placebo (Boele van Hensbroek et al, 1995). In the last study, the participating children, aged between 6 months and 9 years, received either SP (1.25 mg/kg pyrimethamine and 25 mg/kg sulfadoxine) and folic acid (5 mg per day for children weighing < 15 kg, 7.5 mg/kg per day for those weighing 15-20 kg and 10 mg per day for those weighing > 20 mg/kg), SP and iron, or SP and placebo. Parasitological failure rates were 10.5% (9/86) at day 7 among children receiving SP and folic acid, and 3.1% in the other groups combined (5/160, *P* = 0.04); the cumulative failure rate at day 28 was 30.1% (22/73) among the children who received SP and folic, and 16.0% (21/131, *P* = 0.02) among the children in the other groups. The authors suggest that the treatment failures may have been due to *P. falciparum* strains with pyrimethamine resistance, whereby folic acid supplementation made it possible to bypass the sulfadoxine action (Boele van Hensbroek et al, 1995).

More recent in-vitro studies into mechanisms of drug resistance of SP suggest that the interaction may be more complicated and that the synergy between pyrimethamine and sulfadoxine may be affected by folate levels (Wang et al, 1997, 1999). It is suggested that in parasites using exogenous folate efficiently, sulfadoxine inhibition can be restored by pyrimethamine. As the mean half time of sulfadoxine is about twice that of pyrimethamine, their model predicts that, during a period of 3-13 days, when pyrimethamine exceeds a certain blood level, folate utilization will be prevented; this time period will depend upon drug elimination rates and parasite genotype. Once the pyrimethamine concentration drops below this level, any surviving parasites can resume the use of exogenous folate. The degree to which pyrimethamine can inhibit folate utilization could be expected to be dose-dependent and the research group suggest that “loading patients with very large amounts (5-10 mg) of folate daily will inevitably reduce the ability of pyrimethamine to maintain dihydropteroate synthetase as a relevant target for sulfadoxine” (Sims et al, 1999). Prediction of the fate of parasites in individual cases is complicated by host factors, such as the degree of immunity, variation in folate levels and drug metabolism, and parasite factors such as their genotypes involved in drug resistance to SP and their efficiency in using exogenous folate.

**Drug resistance genes and SP**

The development of drug resistance by *P. falciparum* has been attributed to a combination of factors: human migration, heavy malaria transmission and sustained and often haphazard use of drugs. Watkins and Mosobo postulated that selective pressure for resistance is a function of a long elimination half-life of a drug (Watkins & Mosobo 1993). Both components of SP have a long half-life (about 100 hours for pyrimethamine and 200 hours for sulfadoxine), and this may result in the selection of drug resistant mutations at non-inhibitory drug levels during the drug-elimination phase. Simple and reliable methods for drug resistance testing could be provided by molecular assays when is known which genes are involved. Several mutations in parasite DHFR and DHPS have been associated with drug resistance in in-vitro studies, e.g. mutations at codon 108 and 59 for DHFR. In a study in Malawi, infections caused by parasites with 3DHFR mutations and 2DHPS mutations (the quintuple mutant) were associated with SP treatment failure. Although the presence of a single DHFR mutation (Arg-59) with a single DHPS mutation (Glu-540) predicted the presence of the quintuple mutant, this model needs to be validated in other settings (Kublin et al, 2002), making the determination of mutations not yet available as a tool for surveillance of SP resistance (Dicourte et al, 1999).

**HIV and folic acid and SP**

It has been shown repeatedly that HIV-seropositive pregnant women have a higher risk of malaria than HIV-seronegative pregnant women (Steketee *et al*. 1996b, Verhoeff *et al*. 1999). Moreover, a recent study suggested that IPT with SP showed reduced efficacy in clearing placental parasitemia in HIV-seropositive compared to HIV-seronegative pregnant women. However, no difference was observed between the two groups in the clearance of peripheral parasitemia (Parise *et al*. 1998).

HIV-infected pregnant women are more likely to be anemic compared with HIV-uninfected women (van Eijk et al, 2001, Friis et al. 2001). In a recent study in pregnant women in Zimbabwe, HIV-infection was a negative predictor of serum folate, and the authors suggested this may be because of reduced intake and absorption, and increased catabolism in HIV-infected pregnant women (Friis et al, 2001).

Because HIV-seropositive women as a group may have a different folic acid status (and a potential different reaction to folic acid supplementation) than HIV-seronegative women, it will be important to assess HIV-status in study participants It will also be important to confirm that no difference exists between HIV-seropositive and HIV-seronegative women in efficacy of SP for clearance of peripheral parasitemia.

**Sickle cell disease, sickle cell trait and SP**

Sickle cell trait is common among the population in the study area, and was observed in 26% of children participating in an anemia treatment study in an adjacent rural area (M.Desai, personal communication). Sickle cell disease was prevalent in less than 1% of the children participating in the same study. Sickle cell status of participants will be important to include in this study for two reasons:

1) A lower risk of treatment failure with SP for children with sickle cell trait has been reported, so sickle cell trait may act as a confounder in the association between SP treatment failure and folic acid (D. Terlouw, personal communication).

2) Women with known sickle cell disease should receive folic acid supplementation and therefore should be excluded from this study.

**V. Justification**

We propose a placebo-controlled trial to examine the effect of daily folic acid supplementation on efficacy of SP in reducing peripheral parasitemia in pregnant women in western Kenya. Folic acid supplementation is part of recommended care for pregnant women in Kenya. IPT with SP is the Kenya national policy for the control of malaria in pregnancy. However, data exist that suggest that folic acid supplementation in combination with SP treatment for malaria impairs clearance of parasitemia in children. It is therefore important to determine if concomitant folic acid supplementation reduces the efficacy of SP in clearance of *P. falciparum* parasitemia in pregnant women, and if delaying of the intake of folic acid tablets for 2 weeks following an SP treatment dose or reducing the folic acid dose may be a safe alternative. It is important to note that all study women will receive folate supplementation, but in two groups it will be continued when SP is given, while in the other group folate supplementation will be suspended for 2 weeks following the SP treatment dose. Although the Kenya national recommendation is to start folic acid supplementation two days after SP is taken, we are concerned that two days may not be sufficient, given the long half-life of SP.

If concomitant folic acid supplementation (either 5 mg or 400 microgram) is found to impair the efficacy of SP treatment in pregnant women, this will have implications for the Kenya national malaria control guidelines as well as all countries in malaria-endemic areas where IPT with SP during pregnancy is recommended. In Kenya, pending on the results of this study, the delay of folic acid intake after treatment with SP may need to be changed from two days to two weeks or the dose of folic acid may need to be reduced when it is given in combination with SP.

**VI. Objectives**

The purpose of this study is to assess in pregnant women

1. If concomitant folic acid supplementation affects the efficacy of SP in clearance of maternal *P. falciparum* peripheral parasitemia, and improving hemoglobin levels.

2. If SP is still efficacious in treatment of malaria parasitemia in pregnant women in an urban area where SP has been used as first line treatment and for IPT in the past three years.

Secondary objective:

- 1. If HIV infection status affects SP efficacy in clearance of maternal *P. falciparum* peripheral parasitemia.

**VII. Design and Methodology**

**A. Study sites**

This study will be conducted at Nyanza Provincial General Hospital (NPGH), Bondo District Hospital (BDH) and Siaya District Hospital (SDH).

NPGH is a government hospital in Kisumu. Kisumu is located on the shores of Lake Victoria in western Kenya and has about 300 000 inhabitants. Nyanza Provincial General Hospital has been the Ministry of Health site of other CDC/KEMRI studies, including a previous study to assess the safety and efficacy of intermittent SP in the reduction of placental malaria in pregnant women and a study to examine the interaction between malaria in pregnancy and HIV infection. HIV seroprevalence among pregnant women participating in the previous study was 25% and malaria prevalence in the third trimester was 20.1%. This was, however, a selected group of women in their third trimester with uncomplicated pregnancies; women who were excluded may have been more likely to be parasitemic, and women in their second trimester are more likely to be parasitemic compared to women in their third trimester. We expect the malaria prevalence in this ANC population to be higher than 20.1%. Sentinel surveillance in the same hospital has reported an HIV prevalence of 33% among pregnant women (Glynn et al, 2000).

Bondo and Siaya are both located approximately 70 km from Kisumu, in Western Kenya. Bondo has a population of about 30 000 inhabitants (Kenya Malaria Information Service, District Population Projections). BDH is a 42-bed facility, serving the local low-income population. Approximately 400 women attend the ANC per month and one third of them do so for the first time. BDH has collaborated in other CDC/KEMRI studies previously, including a randomized controlled trial of the efficacy of bednets in reducing all cause mortality in Rarieda Division, a nearby area.

The antenatal clinic in SDH sees approximately the same number of women as in Bondo. Siaya hospital is the site where at present a study is conducted assessing the efficacy of treatment with SP among adults with known HIV-status.

In all sites, HIV-counseling and testing is provided in the ANC as part of a program to provide nevirapine to HIV-seropositive pregnant women during delivery to reduce vertical transmission of HIV. In addition, the study clinic employs experienced counselors who can assist with counseling. CDC/KEMRI hospital staffs have been trained to conduct interviews of patients, and to collect and properly handle laboratory samples. The main CDC/KEMRI laboratories are located in Kisian, 10 kilometers from Kisumu.

**B. Design**

We propose a study to look at the interaction between folic acid supplementation and intermittent treatment with SP for malaria in pregnancy in an area where both HIV infection and malaria are common.

We will use a randomized double blind placebo-controlled design, where by pregnant women with peripheral parasitemia will be at random allocated to receive one of three treatment options:

Arm 1: 3 tablets of SP and daily folic acid, 5 mg, according to the national policy (SP/FA5)

Arm 2: 3 tablets of SP and daily folic acid, 400 g, according to international recommendations, to be replaced by folic acid 5 mg after 14 days (SP/FA0.4)

Arm 3: 3 tablets of SP and daily placebo, up to 14 days after the intake of SP, to be replaced by folic acid 5 mg after 14 days (SP/PFA)

Although participating women will be followed up to 28 days after the start of treatment, folic acid 5 mg will be provided until the end of their pregnancy. Women will not be followed further during pregnancy or delivery.

**C. Study population**

All pregnant women who are presenting at the ANC who have not received folate supplementation during the current pregnancy will be eligible for screening for malaria. Only women with a positive blood smear for malaria will be enrolled in the study.

***Criteria for inclusion***

1. Parasitemia with a parasite density of ≥ 500 parasites/microliter
2. Gestational age > 16 weeks and < 35 weeks
3. Willingness to provide blood samples and participate in HIV counseling and testing
4. Available for follow up for the entire study period
5. Hb > 7 g/dl
6. Age 15-45 years

***Criteria for exclusion***

1. Use of folate in the last 4 weeks

2. Gestational age ≤ 16 weeks or ≥ 35 weeks

3. History of an allergy to sulphonamides or other unknown drugs

4. An intake of sulpha-containing drugs or 4-aminoquinolones in the previous month

5. A urine test positive for sulfa-compounds

6. Sickle cell disease

7. Concomitant diseases needing treatment with co-trimoxazole or other sulfa-containing drug

8. Hb ≤ 7 g/dl

9. Severe malaria or any other serious medical condition requiring hospitalization and/or additional treatment. Clinical danger signs of severe malaria include prostration, impaired consciousness, respiratory distress, multiple convulsions, circulatory collapse, pulmonary oedema, abnormal bleeding, jaundice, and hemoglobinuria. Laboratory signs of severe malaria include severe anemia (Hb < 7 g/dl), hypoglycemia, acidosis, hyperlactataemia, hyperparasitaemia (a parasitemia > 100,000 parasites/µl), and renal impairment

**D. Outcome measures and sample size**

Outcome measures in the three arms will be the prevalence of parasitemia at day 2, 3, 7, 14, 21 and 28 following treatment with SP and change in hemoglobin level comparing day 0 (day of SP treatment) to day 28. In line with international criteria the following definitions are used (WHO 2003, see also Annex 5). Early Clinical Treatment Failure (ETF) will be defined as having at least one of the following: (a) the development of danger signs of severe malaria (see above under exclusion criteria 9) on day 1, 2, or 3 in the presence of parasitemia; (b) parasitemia on day 2 higher than the day 0 count; (c) parasitemia on day 3 in the presence of an axillary temperature of ≥ 37.5; or (d) parasitemia on day 3 ≥ 25% of the parasite count on day 0. Late Clinical Treatment Failure (LTF) will be defined as either the presence of parasitemia on any day from day 4 to 28 in the presence of an axillary temperature of ≥ 37.5, OR the development of danger signs after day 3, without previously meeting any of the criteria of ETF. Late parasitological failure is defined as the presence of parasitemia on any day from day 7 to 28, in the presence of an axillary temperature of < 37.5 without previously meeting any of the criteria of ETF or LTF.

A sample size of 600 women, 200 of whom receive SP and folic acid 5 mg, 200 of whom receive SP and folic acid 400 g and 200 of whom will receive SP and placebo folic acid for 2 weeks, will allow us to detect an increase from 5% to 15% parasitological failure rate at day 7, comparing the folic acid placebo arm with the folic acid 5 mg arm and the folic acid 400 g arm, with 80% power and 95% confidence, when taking 25% loss to follow up into account.

**E. Procedures**

***Recruitment***

Prior to screening, the study will be explained to all eligible pregnant women and informed consent will be obtained for both the screening procedures, which include testing for HIV, and sickle cell trait, as well as for the SP-folic acid study with three arms (see also annex 2). We will explain to the woman that a decision will be made on whether she can enter the main placebo controlled SP study with three arms after the study physician has reviewed the results of her screening.

Women will be screened at the antenatal clinic at the NPGH, BDH and SDH. A history of previous and present medication use and a medical history will be taken. Body-temperature and weight and height will be recorded. A urine sample will be examined for the presence of sulfa compounds. A blood sample will be obtained by venipuncture to measure hemoglobin, and to make a thick and thin blood smear. In addition, blood will be taken for HIV-tests, electrophoresis to assess the sickle cell status of the woman, and a serum folic acid level. The HIV-status, sickle cell status, and serum folic acid level will only be determined for women who have been enrolled. For logistical reasons it will not be possible to have the sickle cell test result the same day. It is expected that most women with sickle cell disease will not qualify for enrollment because of their medical history and hemoglobin level. Women who have been enrolled but are shown to have sickle cell disease will be informed as soon as the result is available; this will be the day after enrollment or on the first follow up appointment at day 2. They will be removed from the study and provided folic acid supplementation free of charge for one month. The blood requirements for the HIV-tests and the sickle cell test are minimal, and taking this sample when taking blood for screening will save the pregnant woman an additional venipuncture if she is enrolled.

Before the woman is enrolled, she will be counseled by the Prevention-of-Mother-to-Child-Transmission-of-HIV program in the antenatal clinic. . Post-test counseling will be done by the PMCT program in the prenatal clinic, and supportive counseling will be offered. Because the HIV counseling and testing for the ANC is confidential, a request for the HIV test result of the HIV tests done in the ANC could harm the thrust build up among counselors and ANC-attendees. HIV tests will be repeated by the study laboratory by code, and participants will be informed about the result of the second HIV tests by the study counselor or study nurse during the first follow up appointment on a weekday, and ongoing counseling will be provided. Women who claim that the HIV results of the study are different from the results in the ANC will be offered a Western blot test and a follow up appointment with the study counselor or study nurse about the result.

Decisions on enrollment in any of the three arms will be made by the study physician according to the inclusion and exclusion criteria listed. When the hemoglobin level and the blood-smear result are available to the study physician, and enrollment criteria have been met, the woman will then be allocated to one of the three treatment regimen: SP once and daily folic acid 5 mg, SP once and daily folic acid 400 g for 14 days, or SP once and folic acid placebo for 14 days. Both client and researcher will be blind to the allocation. The intake of SP and the first dose of folic acid 500 mg, folic acid 400 g or folic acid placebo will be supervised in the study clinic. The folic acid 500 mg, folic acid 400 g and the folic acid placebo will be dispensed in a quantity of 14 tablets. Women will be asked to bring the medication whenever they visit the study clinic, to facilitate tablet counts. An address form will be completed to allow the study team to do home visits if a woman does not turn up for her follow up appointment.

Women who are enrolled will be instructed to take the folic acid 400 g, folic acid 5 mg or placebo daily and to come to the clinic if they feel they need medication other than the study medication. All participants will receive iron supplementation as well (ferrous sulphate 200 mg three times per day, clinical guidelines of the Ministry of Health, Kenya). Clients will be specifically asked not to self-administer antimalarials or other drugs, vitamins or minerals during the period of follow up and to return to the study team if they should develop fever, jaundice, skin rash or any complaint for which they might need medical treatment.

After randomization, the study will provide insecticide-treated bed nets to all enrolled women. The rationale for their use is that they reduce the chances of reinfection of enrolled women. This makes is more likely that a recurrence of parasitemia is a recrudescence rather than a reinfection, whereby a recrudescence would be an indication of treatment failure, and a reinfection would not. In addition, several studies, including one recently completed in western Kenya, indicate that insecticide-treated nets confer benefits to women and their neonates; they were associated with reductions in maternal anemia and parasitemia, and an improvement in infant birth weight (Ter Kuile et al, 2003). Women will be informed about the importance of the personal use of a bed net and instructed on how to use the bed net properly.

***Malaria:*** To identify malaria, a thick and thin smear obtained by venipuncture from the women screened or by finger stick from the women who have been enrolled will be stained with Giemsa and examined microscopically.

***HIV-testing:*** HIV-testing will be performed using the test Determine HIV-1/2 (Abott Laboratories, Dainabot Co. Ltd, Tokyo, Japan) and Unigold HIV (for HIV-1 & HIV-2, Trinity Biotech plc, Bray, Ireland), in case of an inconclusive result the rapid test Capillus HIV-1/HIV-2 (Cambridge Diagnostics, Wicklow, Ireland) will be used. These tests are used by the CDC Global Aids Program in the antenatal clinic and for voluntary testing and counseling in the same hospital. If HIV tests results between the ANC and the study tests differ according to the client, the client will be offered a Western Blot test.

***Hemoglobin:*** Hemoglobin will be measured using a Hemocue® hemoglobin detection system (HemoCue AB, Angelholm, Sweden).

***Sickle cell status:*** The HbS genotype of the enrolled women will be assessed using electrophoresis of a red blood cell haemolysate on cellulose acetate plates (Helena

laboratories, USA). Samples will be collected in EDTA microtainers and stored at 4 "C before testing.

***Sulfa-test urine:*** The method of Mount et al (1996), will be used to test the urine for sulfa compounds. In case of a positive urine test for sulfa-compounds the woman will be excluded from the study.

***Follow up:*** Women will be asked to return on day 2, day 3, day 7, day 14, day 21, and day 28 (see annex 2). At these days the malaria smear will be repeated, and women will be questioned on complaints, side effects and medication use. They will be asked to bring the folic acid tablets (either folic acid 400 g, folic acid 5 mg or folic acid placebo) that are remaining to every clinic visit and a tablet count will be done to ensure compliance. Blood will be taken by finger stick for a malaria smear at every follow up date and in case of intercurrent visits. On day 2 or the first follow-up appointment made on a weekday, all clients will see the study counselor or study nurse to be informed about the HIV-test results of the study. At day 7 an additional 1 ml of blood will be taken for serum folate levels to assess adherence. At day 14 and 28, a hemoglobin measurement will be done. Women who do not return for follow up sampling will be visited at home to ask if they are still interested in participating. They will be encouraged to visit the clinic, or blood samples will be taken at home. If a woman develops early or late treatment failure or shows failure to clear parasites during follow up at day 7, day 14, or day 21, she will be treated with quinine, 600 mg three times daily for 7 days, according to the national guidelines. If a woman is parasitemic on day 28, she will receive her next dose of intermittent presumptive treatment with SP if there are no contra-indications (no history of side-effects), otherwise she will also receive quinine. During enrollment, the importance of ensuring their parasitemia is cleared with the alternative drug (quinine) and the need to adhere to their dosing regimen will be emphasized to women. In addition, they will be advised that quinine in therapeutic doses is felt to be safe to take during pregnancy. They will be informed about the potential side-effects of quinine, and advised that these side effects though unpleasant are almost always reversible. Thus, they will be told to continue the medication despite side effects but told to return to clinic for further consultation if they experience serious side effects or if they feel they cannot tolerate the side effects. Women who have a treatment failure on SP and who are treated with quinine will receive a follow-up appointment after 7 days to ensure clearance of parasitemia. Women who have parasitemia on day 7 after the initiation of quinine for SP treatment failure will be offered admission for supervised quinine intake, paid by the SP-folate study (because of the side-effects, many women may not adhere to quinine). Women who refuse admission for supervised quinine therapy will be offered the option of mefloquine treatment. A follow-up appointment after 1 week will be made to ensure clearance of parasitemia after either supervised quinine therapy or mefloquine therapy. If women do not show up for their post-treatment failure visits, they will be visited at home. Mefloquine is mentioned as an alternative drug that can be used for the treatment of malaria in pregnancy in the “Clinical guidelines for diagnosis and treatment of common conditions”, published by the government of Kenya (MOH Kenya 2002).

***Definitions:***

Documented fever will be defined as an axillary temperature of 37.5 ^o^C. Parasitaemia will be defined as the presence of any asexual bloodstage parasite of *Plasmodium falciparum* in a thick smear after inspection of microscopic fields until 300 leucocytes have been counted. A woman will be considered HIV-seropositive if both tests are positive and HIV-seronegative if both HIV-tests are negative. In case of an inconclusive result the rapid test Capillus HIV-1/HIV-2 (Cambridge Diagnostics, Wicklow, Ireland) will be used. Women with the hemoglobin phenotype HbAS will be defined as having sickle cell trait, and women with the hemoglobin phenotype HbSS will be defined as having sickle cell disease. Anemia will be defined as a hemoglobin < 11 g/dl, and severe anemia will be defined as a hemoglobin < 7 g/dl. For analysis a hemoglobin < 8 g/dl, moderate-to-severe anemia, will be considered because studies have indicated that a hemoglobin < 8 g/dl may be an important indicator in association with malaria (Verhoeff et al, 1998). Parasitologic failure and treatment failure will be used as previously described.

**F. Data analysis**

Data will be entered using an optical scanner. Data cleaning will be ongoing during the course of the study. The analysis will be done using SPSS and SAS. Cumulative parasite failure will be calculated by day 3, day 7, day 14, and day 28, stratified by treatment group and compared using a chi-squared test. To assess the effect of HIV-infection, the analysis will be repeated when stratified by HIV-status. Results will be presented first to the Kenya Ministry of Health and will then be presented at national and international conferences and submitted for publication in peer-reviewed journals.

F1. Interim analysis

No definite interim analysis is planned. However, drug resistance rates to SP in Africa are increasing and could potentially reach a point during this study where one would have to consider whether it is appropriate to continue giving SP to pregnant women in a clinical trial.

If overall failure rates (clinical and parasitological) reached 50% at 14 days, we plan a blinded interim analysis by treatment group. These data would be provided to a three-member independent panel (two epidemiologists, one statistician) who would review the data and advise the study team if it appropriate to continue the study. They would take into account such factors as differential efficacy between the treatment arms, as well as power to see a difference given the projected numbers of women still to be enrolled at the time of the interim analysis.

**G. Adverse event reporting**

Anticipated Adverse events: Any untoward medical occurrence in a participant during the trial will be recorded as an adverse event. There are certain events detailed below which are anticipated to have a baseline rate. Every four months, the study team will collate these events and ensure there are no significant differences between the study participants and the expected background rates, stratified by HIV status. These events will be reported in a summary statement as part of the yearly continuation. If the study team recognizes an increase above an expected rate, the information will be provided earlier than this annual report.

The adverse events to be included in this definition are fetal loss, premature delivery, neonatal death if baby born premature or low birth weight.

There is an expected rate of fetal loss after 20 weeks gestation in HIV negative women in this setting of approximately 3%. In literature from Malawi, Uganda and India, in HIV positive women, this rate may vary from equal to the baseline rate in HIV negative patients to six times this expected rate. Prematurity in similar trials occurs in approximately 14% of pregnancies. This rate may be double in HIV positive patients. Low birth weight occurs in approximately 15% of newborns. This rate may again be double in HIV positive mothers. In a previous large study among pregnant women enrolled in their third trimester in the Provincial hospital in Kisumu, hospitalization rates during pregnancy were 4.7% among HIV positive women and 2.5% among HIV negative women; however, these were hospital admission rates recorded at the time of the third trimester among women with a normal uncomplicated pregnancy and no underlying disease, so the hospital admission rates among the total pregnant population attending this ANC can be expected to be higher than this. Among 2301 hospital deliveries in the same hospital recorded between June 1999 and June 2000, there were 12.3% babies born with low birth weight, and 3.7% stillbirths.

Of note, in the current study, designed to assess the efficacy of an antimalarial drug over a 28-day period, women are not routinely followed until delivery. Therefore, delivery information that becomes available will be only among women recently treated for asymptomatic or symptomatic malaria. Because malaria can contribute to all of the aforementioned adverse outcomes (fetal loss, premature delivery, low birth weight, and hospitalization), the rates of these outcomes among women in the study are likely to be higher than rates among the general population of pregnant women.

Unanticipated Adverse Events: A serious unanticipated adverse event will require expedited reporting to the IRB. These will include any maternal death, any serious drug reaction, any fetal death not explained by prematurity or low birth weight or any additional unexpected outcome. These events will be reported to the CDC IRB within 7 days via the adverse event report form provided for by the IRB.

**VIII. Ethical considerations**

**A. Confidentiality**

No study participant will be identified by name in any report or publication derived from information collected for the study. Individual laboratory results will be made available only to the study participant and the clinical personnel at the hospital. All personal identifiers will be removed from the data when it is entered into the computer. Data collection forms will be kept in a secure locked cabinet.

**B. Informed consent**

The purpose of the study, and the risks and benefits will be explained to all potential study participants in the local language. We will clearly communicate that participation in the study is voluntary, that a participant may withdraw from the study at any time, and that access to health care is not dependent on study participation. Only women who provide written informed consent will be enrolled.

The age of majority in Kenya is 18 years. However, many women in sub-Saharan Africa are married and/or running independent households prior to 18 years of age. For example, in our prior work in Kenya, where primigravidae and secundigravidae of any age participated, enrolled women ranged in age from 14-43 years; over one third of the 15-year-olds were married and 71% of enrolled primigravidae and secundigravidae were under 21 years old. In addition, limiting enrollment to only those above the legal age would significantly lower the eligible number of participants and, thus both hamper efficiency of the study and potentially could make the results not extrapolable to all pregnant women. The community would consider women who are less than 18 years old but who are running independent households as emancipated.

Participants aged 15 to 17 years who are married, pregnant, parents, engaged in behavior that puts them at risk or are child sex workers are considered “mature minors” by the Kenyan Ministry of Health (National Guidelines for Voluntary Counseling and Testing (VCT), page 5, paragraph 5) and can give their own consent for VCT. These mature minors will be included in the study, and a waiver for parental or spousal permission is requested under the criteria in section 45CFR46.116(d) and 45CFR46.408(c). This research study involves no more than minimal risk to the study participants; they will receive a drug that is used as part of a national program to control malaria in pregnancy, and will receive a drug that may benefit their health and the health of their baby immediately, or receive this drug after two weeks. The waiver of parental permission will not adversely affect the rights and welfare of the participants. If parental permission were required, participants who test HIV-positive may be at risk of stigma or discrimination by parents, family, or spouse. The cognitive capacity for most 15-17 year olds is similar to adults, so they will be asked to sign the adult consent form and, if appropriate, will be provided with additional pertinent information after participation. All of the study participants will be provided with testing, counseling, and referral for treatment for HIV infection in an entirely confidential manner. However, the choice to include parents, spouse or other family members in the decision whether or not to participate in the study and to be tested for HIV will be left for potential participants to make freely.

The father’s consent need not to be obtained if he is unable to consent because of unavailability, or where the father is, or who he is, is not known for sure, incompetence, or temporary incapacity of the father, or the pregnancy resulted from rape or incest. If the baby’s father is reasonably available, the mother will be asked to have him come to the ANC where he will be given an explanation of the study and all its procedures and asked to sign consent.

Money for transport will be provided to the study participants, to facilitate appearance for follow up visits. The amount of money provided for transport will depend on the distance between their house and the study clinic, and their means of transport (bicycle, public means) and can range from 40 Kenyan shilling to 200 Kenyan shilling for a return journey. It is difficult to make an estimation of the daily wage of the large group of clientele of this particular ANC, many women are housewives, but it is estimated that women may earn between 50 and 400 Kenyan shilling per working day.

***HIV testing***

In all sites, HIV counseling and testing is integrated in the antenatal clinic routine as part of a program to reduce vertical transmission of HIV by providing nevirapine during delivery. Women identified as HIV-positive will be offered nevirapine through the prevention of mother-to-child transmission (PMCT) program of the hospital. Ongoing medical support for HIV-seropositive women and their children is available. HIV-positive women can be referred for ongoing medical support to the Patient Support Center of the Provincial hospital. Women who meet WHO criteria for ARV treatment are referred to the PMCT+ program in the Provincial hospital that can provide ARV treatment.

The laboratory tests for the study will be conducted by code, and not by name, and HIV laboratory results will be entered in the computer by code only. All women who are enrolled will meet with the study counselor or study nurse on the first follow up appointment on a weekday, to be informed about the HIV test results of the HIV tests done for the study, and ongoing counseling will be provided. In addition, the sickle cell test results will be given. If women claim the study HIV test results are different from the HIV test results in the ANC, a Western blot test will be offered. The study doctor may occasionally retrieve the HIV-status of a client when it is considered important for the clinical treatment of the person.

**C. SP in pregnancy**

SP has been used during pregnancy for both treatment and prophylaxis of malaria. No adverse pregnancy outcomes have been reported in several studies. Although there is a theoretical risk of kernicterus in the newborn if prenatally exposed to SP late in gestation (because sulfa compounds can supposedly interfere with fetal bilirubin metabolism), in reality there is no evidence that this is an important clinical problem. The two-dose regimen of SP for prevention of malaria during pregnancy has been adopted by the Kenya National Malaria Control Programme and implemented in the Provincial hospital in March 1999.

**D. Benefits to study participant**

The dose of SP will treat malaria in the study participant and might provide protection against delivering a low birth weight infant through clearance of placental parasitaemia. The dose of SP will decrease the chance of anaemia. Treatment of malaria will be provided free of charge to the participant during the study period. Folic acid tables and iron tablets will be available for the participating women free of charge for the duration of the study. Contact with the counselor is an opportunity for the women to become better educated about HIV. The results of the electrophoresis test will be made available to the participating women and an opportunity will be created to consult a local specialist in sickle cell disease (Dr Juliana Otieno). Enrolled women will receive an impregnated net to protect themselves from malaria-reinfection. Because women have to prepare themselves for the delivery, they will be provided with a babykit containing clothes, a towel and a babysheet, and a pot of Vaseline. The babykit is a donation of the National Counsel of Churches for this purpose.

**E.** **Risks to the study participant**

Women who will be in the placebo group will not receive folic acid for two weeks, however, just missing two weeks of folate should not have a major effect on anemia (Fleming et al, 1968), and temporarily interrupting supplementation after SP therapy may improve parasite clearance and thus providing the benefit of fully effective antimalarial therapy. In addition, treatment of malaria in primigravidae stabilized serum folic acid levels in a study in Nigeria (Fleming et al, 1968). The issue of the risk of neural tube defects in the absence of folate supplementation during the first trimester will not arise since these women will not be enrolled until the second or third trimester of pregnancy. Women who receive folate (either folic acid 5 mg or folic acid 400 g) during the study period may be at an increased risk of treatment failure, and they will be carefully followed. Previous studies in pregnant women have not shown an increased risk of treatment failure in women prescribed folic acid, but these studies were not specifically designed to address this issue and compliance with folic acid was not monitored. All participating women will receive iron tablets during the study period.

When a pregnant woman is screened, 1.5 ml of blood is needed for the measurement of hemoglobin, a blood smear for malaria, HIV testing, serum folic acid levels and sickle cell testing. The total amount of blood that is needed is approximately 3 ml, over a period of 4 weeks. The amount of blood cells involved is easily replaced by blood cells from the reservoir in the bone marrow in pregnant women and the volume of fluid by fluid intake per os. Severely anaemic women (those with a hemoglobin equal to or less than 7 g/dl) will be excluded from the study and referred for treatment. For the collection of blood samples new disposable lancets will be used.

There are no indications from the previous studies in pregnant women in Malawi and Kenya that intermittent use of SP treatment doses (once per trimester or once monthly) was associated with severe skin reactions as has been reported for weekly prophylactic use (Parise et al, 1998, Verhoeff et al, 1998). The Kenyan guidelines for IPT will be followed, excluding the delivery of SP to pregnant women in their first trimester, and thus the potential teratogenic effect of pyrimethamine and sulfadoxine, and excluding the delivery of SP to women after 34 weeks of gestational age, and thus the theoretical risk of kern-icterus in the newborn in association with sulphonamides (Phillips-Howard & Wood, 1996).

Pregnant women identified with sickle cell disease who have been enrolled in the study will be contacted as soon as possible, either before or at their scheduled appointment on day 2. They will be provided with folic acid and excluded from the study. If they have been enrolled in the “no-folate” arm of the study, this means there will be a delay of one or two days before they start on folic acid. We do not expect that the deprivation of folic acid for one or two days will have serious detrimental effects on their health. However, we anticipate that, given the range of complaints associated with sickle cell disease in this environment and the likelihood of anemia, most women with sickle cell disease will have been excluded from participating in this study during the screening phase.

Pregnant women who do not want to participate in the study, will receive the two-dose regimen of SP, and will be given folic acid tablets free of charge in the ANC provided by the Ministry of Health.

Women with a treatment failure to SP will be treated with quinine and followed after 1 week to ensure clearance of the parasitemia. Women who do not clear their parasitemia will be offered admission for daily supervised quinine treatment free of charge. Women who refuse admission will be given the option of taking mefloquine after an explanation of potential risks and benefits. All women with parasitemia after quinine treatment will get a follow up appointment 1 week after the alternative treatment until their parasitemia has cleared.

The most common side effects reported with quinine therapy include dizziness, decreased hearing, tinnitus, blurred or double vision, photophobia, nausea, and vomiting. Although the US drug labeling categorizes quinine (Merrell Dow) as Category X (contraindicated), the congenital abnormalities reported occurred at abortifacent doses (Briggs et al, 1986). In addition, quinine at therapeutic doses is not felt to induce premature delivery (Looareesuwan et al, 1985). Quinine is recommended as the drug of choice for treatment of uncomplicated and severe malaria during pregnancy, including CDC (http://www.cdc.gov/malaria/diagnosis_treatment/clinicians3.htm), Health Canada (CATMAT: http://www.hc.sc.gc.ca/pphb-dgspsp/publicat.htm) and the WHO (WHO 2001).

The most common potential adverse drug reactions associated with mefloquine include headache, nausea, dizziness, difficulty sleeping, anxiety, vivid dreams, and visual disturbances. Mefloquine treatment can also cause serious side effects, such as seizures, depression, and psychosis. Mefloquine has not been shown to be associated with congenital abnormalities (Phillips-Howard & Wood, 1996; McGready et al 2002). However, one retrospective study from Thailand reported that mefloquine treatment may be associated with an increased rate of stillbirths but no definite conclusions could be drawn (WHO 2001, Nosten et al 1994, 1999). Given this uncertainty, we feel that mefloquine should be used only for women who fail quinine therapy (or who cannot tolerate it). Because malaria can have severe adverse consequences for both mother and fetus, women need treatment for parasitemia during pregnancy and we feel that in women who have failed quinine (who have no other better option), the benefits of mefloquine therapy outweigh the risks. WHO has recommended that mefloquine can be used for treatment of malaria during pregnancy in the 2^nd^ and 3^rd^ trimesters (WHO 2001).

Although amodiaquine is regularly used in Kenya for malaria in pregnancy, and is mentioned as well as a treatment option in the national guidelines (MOH 2002), a recent review exposed the lack of safety data on amodiaquine in pregnancy (Thomas et al, 2004), and for this reason we do not feel it is a good option for treatment of SP treatment failure.

The protocol will be submitted for scientific and ethical clearance to the Institutional Review Board of KEMRI and CDC. No specimens will be stored for possible future use. Expedited review is requested because this is a minimal risk study and the methods of data collection fall under categories: (1) Clinical studies of drugs for which an IND is not required; (2) Collection of blood samples by venipuncture; and (3) Prospective collection of biological specimens (urine)

by noninvasive means.

**IX. Research team**

This study will be a collaborative project between the Nyanza Provincial General Hospital, Kisumu, Bondo District hospital, and Siaya District hospital, hospitals of the Ministry of Health of Kenya, and the VBCRC KEMRI-CDC, based in Kisumu. The work in the hospitals (enrollment and follow up) will be conducted under the clinical supervision of Dr. van Eijk, Physician and Research Officer, Mr. Ayisi (Research Officer), Dr. Peter Ouma (Research officer), Dr. Mary Hamel (Research Physician), Dr Otieno, the study physician and Dr Odondi, the Medical Superintendent. The laboratory work will be supervised by Dr Shi. Data analysis and reporting will be conducted in collaboration with Dr. ter Kuile (epidemiologist), Dr. Slutsker (Director Kisumu field station), Dr. Parise (epidemiologist) and Dr. Misore (Provincial Medical officer). Data entry and statistical analyses will be supervised by Dr. Rosen (epidemiologist). Dr. van Eijk (CDC/KEMRI) and Dr. Slutsker will be the overall coordinators and principal investigators.

**X. Proposed Time Frame**

1. **Data collection**. Preparation of the study and testing of forms will start in June 2003. Recruitment of pregnant women will start in July 2003 and continue for 31 months. Sample collection of pregnant women will be finished in 30 months.

2. **Data analysis and report preparation**. Sample analysis and data analysis are planned to be completed by the end of 2006. The report will be completed and submitted by the beginning of 2007.

**Disseminating results to public**

The results of the study will be discussed with the Provincial Medical Officer, and the national malaria control program. They will then be presented at national and international conferences and submitted for publication in peer-reviewed journals.

**XII. REFERENCES**

Boele van Hensbroek M, Morris-Jones S, Meisner S, Jaffar S, Bayo L, Dackour R, Phillips C, Greenwood BM, 1995. Iron, but not folic acid, combined with effective antimalarial therapy promotes haematological recovery in African children after acute falciparum malaria. *Trans R Soc Trop Med Hyg 89:* 672-676.

Brabin BJ, 1983. An analysis of malaria in pregnancy in Africa. *Bull World Health Organ 61:* 1005-1016.

Brabin BJ, van den Berg H, Nijmeyer F, 1986. Folacin, cobalamin, and hematological status during pregnancy in rural Kenya: the influence of parity, gestation, and Plasmodium falciparum malaria. *Am J Clin Nutr 43:* 803-815.

Bradley-Moore AM, Greenwood BM, Bradley AK, Akintunde A, Attai ED, Fleming AF, Flynn FV, Kirkwood BR, Gilles HM, 1985. Malaria chemoprophylaxis with chloroquine in young Nigerian children. IV. Its effect on haematological measurements. *Ann Trop Med Parasitol 79:* 585-595.

Briggs GC, Freeman RK, Yafte SE, 1986. Drugs in pregnancy and lactation. 2^nd^ edition. Baltimore: Williams and Wilkins, 537-553.

Brockelman CR, Tan-ariya P, Piankijagum A, Matangkasombut P, 1983. An in vitro approach to study innate resistance to Plasmodium falciparum infection in folic acid deficient individuals. *Asian Pacific J Allerg Immun 1:* 107-111.

Centers for Disease Control, 1992. Recommendations for the use of folic acid to reduce the number of cases of spina bifida and other neural tube defects. *MMWR 41:* RR-14.

Dicourte Y, Djimde A, Duombo OK, Sagara I, Coulibaly Y, Dicko A, Diallo M, Diakite M, Cortese JF, Plowe C, 1999. Pyrimethamine-sulfadoxine efficacy and selection for mutations in *Plasmodium falciparum* dihydrofolate reductase and dihydropteroate synthase in Mali. *Am J Trop Med Hyg 60:* 475-478.

Fleming AF, Hendrickse JP, Allan NC, 1968. The prevention of megaloblastic anaemia in pregnancy in Nigeria. *J Obstet Gynaec Brit Cwlth 75:* 425-432.

Food and Nutrition Board of the Institute of Medicine, 1999. Dietary Reference Intakes for Thiamin, Riboflavin, Niacin, Vitamin B6, Folate, Vitamin B12, Pantothenic Acid, Biotin, and Choline. Washington DC: The National Academy of Sciences.

Friis H, Gomo E, Koestel P, Ndhlovu P, Nyazema N, Krarup H, Fleisher Michaelsen K, 2001. HIV and other predictors of serum folate, serum ferritin, and hemoglobin in pregnancy: a cross-sectional study in Zimbabwe. *Am J Clin Nutr 73:* 1066-1073.

Fuller NJ, Bates CJ, Hayes RJ, Bradley AK, Greenwood AM, Tulloch S, Greenwood BM, 1988. The effects of antimalarials and folate supplements on haematological indices and red cell folate levels in Gambian children. *Ann Trop Med Paediatrics 8:* 61-67.

Gail K, Herms V, 1969. Der Einfluss von pteroylglutaminsaure (“Folsaure”) auf die parasitendichte (Plasmodium falciparum) bei schwangeren Frauen in West-Afrika. *Z Tropenmed Parasit 20:* 440-450.

German Nutrition Society. Die Referenzwerte fur die Nahrstoffzufuhr. Die Deutsche Gesellschaft fur Ernahrung e.V., die Osterreichische Geselschaft fur Ernahrung, die Schweizerische Gesellschaft fur Ernahrungsforschung, die Scheweizerische Vereinigung fur Ernarhrung. Frankfurt am Main, 2001.

Glynn JR, Buve A, Carael M, Kahindo M, Macauley IB, Musonda RM, Jungmann E, Tembo F, Zekeng L, 2000. Decreased fertility among HIV-1-infected women attending antenatal clinics in three African cities. *J Acquir Immune Defic Syndr 25:* 345-352.

Hercberg S, Chauliac M, Galan P, Devanlay M, Zohoun I, Agboton Y, Soustre Y, Bories C, Christides J-P, Potier de Courcy G, Masse-Raimbault A-M, Dupin H, 1986. Relationship between anaemia, iron and folacin deficiency, haemoglobinopathies and parasitic infection. *Hum Nutr Clin Nutr 40:* 371-379.

Higgins JR, Quinlivan EP, McPartlin J, Scott JM, Weir DG, Darling MRN, 2000. The relationship between increased folate catabolism and the increased requirement for folate in pregnancy. *Br J Obstet Gynaecol 107:* 1149-1154.

Hughes A, Gatus BJ, 1979. Severe megaloblastic anaemia caused by Maloprim. *J Trop Med Hyg 82:* 120-121.

Hurley MGD 1959. Administration of pyrimethamine with folic acid and folinic acids in human malaria. *Trans R Soc Trop Med Hyg 53:* 410-411.

Kaseje DCO, Sempebwa EKN, Spencer HC, 1987. Malaria chemoprophylaxis to pregnant women provided by community health workers in Saradidi, Kenya. I. Reasons for non-acceptance. *Ann Trop Med Parasitol 81 (suppl 1):* 77-82.

Kublin JG, Dzinjalama FK, Kamwendo DD, Malkin EM, Cortese JF, Martino LM, Mukadam RAG, Rogerson SJ, Lescano AG, Molyneux ME, Winstanley PA, Chimpeni P, Taylor T, Plowe CV, 2002. Molecular markers for failure of sulfadoxine-pyrimethamine and chlorproguanil-dapsone treatment of *Plasmodium falciparum* malaria. *J Infect Dis 185:* 350-358.

Looareesuwan S, Phillips RE, White NJ, Kietinun S, Karbwang J, Rackow C, Turner RC, Warrell DA, 1985. Quinine and severe falciparum malaria in late pregnancy. *Lancet 2(8445):* 4-8.

Lumley J, Watson L, Watson M, Bower C, 2001. Periconceptual supplementation with folate and/or multivitamins for preventing neural tube defects (Cochrane review). *Cochrane Database Syst Rev 3:* CD001056.

Mahomed K, 2000. Folate supplementation in pregnancy. *Cochrane Database Syst Rev (2):* CD000183.

McCormick MC, 1985. The contribution of low birth weight to infant mortality and childhood morbidity. *N Engl J Med 312:* 82-90.

McGready R, Thwai KL, Cho T, Looaresuwan S, White NJ, Nosten F, 2002. The effects of quinine and chloroquine antimalarial treatments in the first trimester of pregnancy. *Trans R Soc Trop Med Hyg 96:* 180-184.

Menendez C, 1995. Malaria during pregnancy: A priority area of malaria research and control. *Parasitol Today 11:*178-183.

Milhous WK, Weatherly NF, Bowdre JH, Desjardins RE, 1985. In vitro activities of and mechanisms of resistance to antifol antimalarial drugs. Antimicrob Agents Chemother 27: 525-530.

Ministry of Health, Government of Kenya, 1994. Clinical guidelines for diagnosis and treatment of common hospital conditions in Kenya. Nairobi, Kenya.

Ministry of Health, Kenya, 2000. A simplified national guideline on malaria control for community resource persons. [Http://www.kmis.org/guide.htm](http://www.kmis.org/guide.htm)

Ministry of Health, Government of Kenya, 2002. Clinical guidelines for diagnosis and treatment of common conditions. October 2002, Nairobi, Kenya

Mount DL, Green MD, Zucker JR, Were JBO, Todd GD, 1996. Field detection of sulfonamides in the urine: the development of a new and sensitive test. *Am J Trop Med Hyg 55:* 250-253.

National Academy of Sciences. Dietary reference intakes for thiamin, riboflavin, niacin, vitamin B6, folate, vitamin B12, panthotenic acid, biotin and choline (1999). [Http://www.nap.edu/open](http://www.nap.edu/open) book/0309065542/html/196.html copyright 1999, 2000.

Nosten F, ter Kuile F, Maelankiri L, Chongsuphajaisiddhi T, Nopdonraakoon L, Tangkitchot S, Boudreau E, Bunnag D, White NJ, 1994. Mefloquine prophylaxis prevents malaria during pregnancy: a double-blind, placebo-controlled study. *J Infect Dis 169:* 595-603.

Nosten F, Vincenti M, Simpson J, Yei P, Thwai KL, De Vries A, Chongsuphajaisiddhi T, White NJ, 1999. The effects of mefloquine treatment in pregnancy. *Clin Infect Dis 28:* 808-815.

Parise ME, Ayisi JG, Nahlen BL, Schultz LJ, Roberts JM, Misore A, Muga R, Oloo AJ, Steketee RW, 1998. Efficacy of sulfadoxine-pyrimethamine for prevention of placental malaria in an area of Kenya with a high prevalence of malaria and human immunodeficiency virus infection. *Am J Trop Med Hyg 59:* 813-822.

Phillips-Howard PA & Wood D, 1996. The safety of antimalarial drugs in pregnancy. *Drug safety 14:* 131-145.

Rogerson SJ, Chaluluka E, Kanjala M, Mkundika P, Mhango C, Molyneux ME, 2000. Intermittent sulfadoxine-pyrimethamine in pregnancy: effectiveness against malaria morbidity in Blantyre, Malawi, in 1997-99. Trans R Soc Trop Med Hyg 94: 549-543.

Rieckmann K, Suebsaeng L, Rooney W, 1987. Response to Plasmodium falciparum infections to pyrimethamine-sulfadoxine in Thailand. *Am J Trop Med Hyg 37:* 211-216.

Schultz LJ, Steketee RW, Macheso A, Kazembe P, Chitsulo L, Wirima JJ, 1994. The efficacy of antimalarial regimens containing sulfadoxine-pyrimethamine and/or chloroquine in preventing peripheral and placental Plasmodium falciparum infection among pregnant women in Malawi. *Am J Trop Med Hyg 51:* 515-522.

Shulman CE, Dorman EK, Cutts F, Kawuondo K, Bulmer JN, Peshu N, Marsh K, 1999. Intermittent sulphadoxine-pyrimethamine to prevent severe anaemia secondary to malaria in pregnancy: a randomized placebo-controlled trial. *Lancet 353:* 632-636.

Sims P, Wang P, Hyde JE, 1999. Selection and synergy in *Plasmodium falciparum*. *Parasitology Today 15:* 132-134.

Smits LJM, Essed GGM, 2001. Short interpregnancy intervals and unfavourable pregnancy outcome: role of folate depletion. *Lancet 358:* 2074-2077.

Steketee RW, Wirima JJ, Hightower AW, Slutsker L, Heymann DL, Breman JG, 1996a. The effect of malaria and malaria prevention in pregnancy on offspring birthweight, prematurity, and intrauterine growth retardation in rural Malawi. Am J Trop Med Hyg 55(1 Suppl): 33-41.

Steketee RW, Wirima JJ, Bloland PB, Chilima B, Mermin JH, Chitsulo L, Breman JG, 1996b. Impairment of a pregnant woman’s acquired ability to limit *Plasmodium falciparum* by infection with human immunodeficiency virus type-1. *Am J Trop Med Hyg 55 (suppl):* 42-49.

Steketee RW, Nahlen BL, Parise ME, Menendez C, 2001. The burden of malaria in pregnancy in malaria-endemic areas. *Am J Trop Med Hyg 64(1-2)S:* 28-35.

Stoltzfus RJ, Dreyfuss ML, 1997. Guidelines for the use of iron supplements to prevent and treat iron deficiency anemia. International Nutritional Anemia Consultative Group, ILSI press, Washington D. C. USA.

Sturchler D, Holzer B, 1980. Low serum folate among persons raking Fansidar (pyrimethamine plus sulfadoxine) for prophylaxis of malaria. Acta Trop 37: 243-248.

Tan-Ariya P, Brockelman CR, 1983. *Plasmodium falciparum:* variations in *p*-aminobenzoic acid requirements as related to sulfadoxine sensitivity. *Exp Parasitol 55:* 364-371.

Ter Kuile FO, Terlouw DJ, Phillips-Howard PA, Hawley WA, Friedman JF, Kariuki SK, Shi YP, Kolczak MS, Lal AA, Vulule JM, Nahlen BL, 2003. Reduction of malaria during pregnancy by permethrin-treated bed nets in an area of intense perennial malaria transmission in Western Kenya. *Am J Trop Med Hyg 68 (Suppl 4):* 50-60.

Thomas F, Erhart A, D’Alessandro U, 2004. Can amodiaquine be used safely during pregnancy? *Lancet Infect Dis 4:* 235-239.

Tong MJ, Strickland GT, Votteri BA, Gunning JJ, 1970. Supplemental folates in the therapy of Plasmodium falciparum malaria. *JAMA 214:* 2330-2333.

Trager W, 1959. The enhanced folic and folinic acid contents of erythrocytes infected with malaria parasites. *Exp Parasitol 8:* 265-273.

United Nations Children’s Fund, 1998. *The State of the World’s Children*. New York: Oxford University Press.

Verhoeff FH, Brabin BJ, Chimsuku L, Kazembe P, Russell WB, Broadhead RL, 1998. An evaluation of the effects of intermittent sulfadoxine-pyrimethamine treatment in pregnancy on parasite clearance and risk of low birth weight in rural Malawi. *Ann Trop Med Parasitol 92:* 141-150.

Verhoeff FH, Brabin BJ, Hart CA, Chimsuku L, Kazembe P, Broadhead RL, 1999. Increased prevalence of malaria in HIV-infected pregnant women and its implication for malaria control. *Trop Med Int Health 4:* 5-12.

Verhoeff FH, Brabin BJ, Chimsuku L, Kazembe P, Broadhead RL, 1999. An analysis of the determinants of anaemia in pregnant women in rural Malawi – a basis for action. *Ann Trop Med Parasitol 93:* 119-133.

Wald NJ, Law MR, Morris KJ, Wald DS, 2001. Quantifying the effect of folic acid. *Lancet 358:* 2069-2073.

Wang P, Read M, Sims PFG, Hyde JE, 1997. Sulfadoxine resistance in the human malaria parasite Plasmodium falciparum is determined by mutations in dihydropteroate synthetase and an additional factor associated with folate utilization. *Molecular Microbiology 23:* 979-986.

Wang P, Brobey RKB, Horii T, Sims PFG, Hyde JE, 1999. *Molecular Microbiology 32:* 1254-1262.

Watkins WM, Sixsmith DG, Chulay JD, Spencer HC, 1985. Antagonism of sulfadoxine and pyrimethamine antimalarial activity in vitro by p-aminobenzoic acid, p-aminobenzoylglutamic acid and folic acid. *Mol Biochem Parasitol 14:* 55-61.

Watkins WM, Mosobo M, 1993. Treatment of *Plasmodium falciparum* malaria with pyrimethamine-sulfadoxine: selective pressure for resistance is a function of long elimination half-life. *Trans R Soc Trop Med Hyg 87:* 75-78.

Weidekamm E, Plozza-Nottebrock H, Forgo I and Dubach UC,1982. Plasma concentrations of pyrimethamine and sulfadoxine and evaluation of pharmacokinetic data by computerized curve fitting. *Bull WHO 60:* 115-121.

WHO expert committee on malaria, 2000. Twentieth report. *WHO technical report series 892*, World Health Organization, Geneva.

WHO 2001. The use of antimalarial drugs. Report of an informal consultation. World health organization, Geneva.

WHO 2003. Assessment and monitoring of antimalarial drug efficacy for the treatment of uncomplicated malaria. WHO/HTM/RBM/2003.50 Geneva.

Annex 1: Folic acid pathway in mammals and bacteria/protozoa

Folic acid is required for DNA synthesis in both humans and bacteria/protozoa. Humans can use folic acid from the diet, and have evolved a transport mechanism for taking it up in the cell. In contrast to the malaria protozoa, humans cannot synthesize folic acid. Most species of bacteria, and the asexual form of malaria protozoa cannot make use of preformed folic acid; they must synthesize their own.

^1^ Food and Nutrition Board, Institute of Medicine, 1999. Dietary Reference Intakes for Thiamin, Riboflavin, Niacin, Vitamin B6, Folate, Vitamin B12, Panthothenic Acid, Biothin and Choline. Washington DC: The National Academy of Science.

Abbreviations: GMP: guanosine monophosphate

This schedule is an over simplification: folic acid is known to interfere with the activity of some sulfonamides in in-vitro malaria models (McCormick & Canfield 1972, Milhous 1983, Watkins 1985). Possible explanations for this are that:

* Dihydropteroate synthase can use para-aminobenzoylglutamate (PABG) as an alternative substrate to form dihydrofolate, although the affinity to PABG is lower than to PABA. The PABG could arise from the cleavage of folates.

*Folic acid may act in another way (e.g. by inhibiting sulfadoxine transport into the cell).

*Folic acid in the human erythrocytes can be directly used by *P. falciparum*

Literature:

Wernsdorfer WH, McGregor I, ed. 1988. Malaria: principles and practice of malariology, volume 2 Churchill Livingstone, Langman Group UK Limited.

Annex 2: Follow up schedule for participating pregnant women

| Day | Action | Form | Laboratory |
| --- | --- | --- | --- |
| 0 | a) Screening  b) Enrollment  c) Randomization  d) Supervised SP  e) Instruction and supply of folic acid/placebo tablets, supervised first dose  f) Supply of iron tablets 60 mg/day | Screening form  Enrollment form | Thin and thick blood smear  Hemoglobin  HIV-testing  Sickle cell test  Filter paper sample for PCR  Urine for sulfa test |
| 2 | a) Follow up,  b) Supervised folic acid/placebo | Follow up form | Thin and thick smear |
| 3 | a) Follow up  b) Supervised folic acid/placebo | Follow up form | Thin and thick smear |
| 7 | a) Follow up  b) Supervised folic acid/placebo | Follow up form | Thin and thick smear |
| 14 | a) Follow up  b) New supply folic acid tablets for all women | Follow up form | Thin and thick smear  Hemoglobin |
| 21 | a) Follow up  b) Supervised folic acid | Follow up form | Thin and thick smear  Filter paper sample for PCR |
| 28 | a) Follow up  b) New supply folic acid /iron | Follow up form | Thin and thick smear  Hemoglobin |
| x | Intercurrent visit: visit in between follow up visits | Follow up form | Thin and thick smear  Hemoglobin |

Annex three: Consent form for women 18 years and mature minors (Flesch-Kincaid level 7.4; the reading level was assessed using WordPerfect version 9, and institution names and contact information were not included.)

**Consent form for the study of the effect of folic acid on efficacy of Fansidar in pregnant women.**

Introduction

The Kenya Ministry of Health, New Nyanza Provincial Hospital, Bondo District hospital, Siaya District hospital and the Centers for Disease Control and Prevention (Atlanta, Georgia, USA) are doing a research study to see if Fansidar (sulfadoxine-pyrimethamine: SP) and folic acid can be given at the same time to pregnant women. When you are pregnant, you are more likely to get malaria. Sometimes you can have it without feeling sick. It can cause your baby to be born small and weak. As you know, the Kenyan government advises pregnant women to use SP to treat and prevent malaria. Pregnant women also need folic acid and iron tablets to stay healthy. These mineral supplements help to make enough blood in the body for the mother and the baby.

Purpose of the research

SP fights malaria in the body by stopping the malaria to use folic acid. When a woman takes folic acid tablets at the same time, it can happen that SP cannot fight the malaria well. We want to see if SP works the same in pregnant women who use or do not use folic acid. We also want to see if it works the same in women with or without the HIV-virus. To do this we need to check the blood of pregnant women who have taken SP for malaria during one month. The study results will help us find out if you can use SP and folic acid tablets at the same time.

Procedures

If you agree to be in the study, we will ask you some questions about your health and the use of drugs in the past months. We will check your weight and if you have fever. This will take about 10 minutes. We will then take a blood sample by venipuncture from your arm. The amount will be 1.5 ml (less than half a teaspoon). This blood will be used to see if you have enough blood, and to check for malaria. A small amount of blood we will keep for HIV-tests, for a sickle cell test and to find out how much folic acid you have in your blood if we find you can be in the follow-up study. We will ask for a sample of urine from you to check for malaria drugs if you are in the follow-up study.

We will ask 600 women to be in the follow-up study. Whether you are asked to be in the follow-up study will depend on the blood test result for malaria. The study doctor will see if you can be in the follow-up study. We will repeat the HIV tests for the follow-up study, but only by code. We want to make sure that the HIV test result is between you and the HIV worker who is trained for this. We will ask you when you come back again on the first weekday to talk with the study HIV worker or the study nurse to get the result of the tests for the follow-up study. No matter what the result is, the result of the blood test for the HIV virus will only be known to you, the study counselor and the study doctor or nurse. The study doctor may need to know it for medical treatment. We will not tell any other person the result unless you ask us to do so.

If you are asked to be in the follow-up study, we will give you three tablets of SP and one other tablet to swallow. This tablet will be a folic acid tablet with a high dose (5 mg), a folic acid tablet with a low dose (0.4 mg) or just look like a folic acid tablet but have no folic acid tablet in it. We will not know which tablet you will receive. We will give you folic acid tablets or tablets that look like folic acid for 14 days. You need to take one tablet everyday. After 14 days you will get new folic acid tablets. This time every woman will receive high dose folic acid tablets. We will ask you to come to the study clinic after two days, three days, seven days, 14 days, 21 days and 28 days. On these days we will check the blood for malaria by a finger prick. On day 7 we will take blood to find out if there is more folic acid in the blood. On day 14 and 28 we will also check the blood level again. If you have fever or the malaria is not going away from your blood, we will give you treatment free of charge (Quinine tablets). Talking to you and taking the blood sample will take about 10 minutes on these days. We will help with money for the transport to the clinic. If you do not come for the blood samples, a member of the study team will come to your home to see if you still want to be in the study. If you are in the follow-up study, we will give you a treated bed net to make the chances lower that you get malaria again.

Your answers to all questions will be kept private as much as the law allows. Only the persons doing the study will know about them. You can refuse to answer any question that you do not like. Your specimens will be destroyed at the end of the study.

Because the treatment and tests you will receive can affect not only you but also your baby, we will ask for the father’s consent as well, if the father is around and knows that you are pregnant. This is because the baby did not come just from you, but also from the father.

If you are in the follow-up study, we will also ask you not to take any other drug than what the team gives you. You can come to the clinic if you feel ill. You will be checked by a clinical officer or the study doctor free of charge who will see if you need more treatment. If she thinks you need a drug that may also affect SP or folic acid, you cannot be in the follow-up study anymore. You also cannot be in the follow-up study if you have sickle cell disease, because women with sickle cell disease should always take folic acid.

Whether you join in the study or not, you should still come to clinic for your health and your baby's health. If you choose not to join in the study, it will not affect the care given to you by the clinic or the hospital. You will get the standard treatment from the ANC. For women like you who are pregnant and in the second or third term, the standard treatment to prevent malaria is one dose of SP now, and one dose of SP in the last term. You will get the routine iron and folic acid tablets in the clinic, and you should not take folic acid for two days when you take SP. If you think you may have malaria, you can still get checked for that at the clinic. If you cannot be in the study, we will not keep your blood for any tests.

Benefits and risks

There will be a slight sting that lasts for a few seconds when we prick your finger to collect blood. In the whole study you only need to give a total amount of 3 ml of blood (a bit more than a tea spoon).

SP will treat your malaria. You are less likely to have too little blood (anaemia) when you have taken a treatment dose of this drug. Studies have shown that SP is safe for you to take when you are pregnant. It will not harm your baby. We believe that treating you for the malaria outweighs any risk from the drug. Rarely, SP and other sulfa drugs may cause skin rash or sores in the mouth, which can be severe. This may happen in one person of 5,000 who receive the drug. If you have ever had a skin rash after taking a drug, please tell us. If you have any side effect that you think might be due to SP, please come back to the clinic right away to be checked and treated by one of the doctors. If it is not during working hours, you have to go to the casualty department of this hospital who will inform us.

If you have side effects, you should not take SP again. You should also tell any health care workers who are treating you in the future about the side effects you have had after taking it. If you need treatment with a drug that can make the testing for malaria in the blood difficult we will stop taking the blood. We will also make sure you get the proper treatment free of charge.

If the SP does not cure your malaria, you will be treated with Quinine. Quinine is the safest drug that we know to treat your malaria if SP does not work. Quinine can cause you to have side-effects that make you feel bad such as feeling dizzy, having problems hearing or seeing, or nausea. These side effects just about always go away when the treatment is done. You have to make sure you take all the tablets that we give you to make it work best. If you start the quinine and start to feel really bad and don’t feel you can continue to take the drug, please come back to the clinic right away and we will help you find another option. If you think you cannot take all quinine pills at home, we can pay for you to go in the hospital and see that you take it.

We will ask you to come back seven days after quinine treatment to check if the malaria has gone. If you do not wish to take quinine, or quinine makes you feel ill after you have started it, we can offer you mefloquine. Mefloquine is a good drug for the treatment of malaria. We prefer not to use it when you are pregnant unless we don’t have a choice. It may affect the baby. In one study stillbirths were more common among women treated with mefloquine than among women who got other treatment. This is why we prefer you take the quinine the way we tell you. However, we think that treating the malaria outweighs the risk of the mefloquine. The Kenyan government guidelines do use mefloquine to treat malaria in pregnant women. The most common side effects that can occur with mefloquine are headache, nausea, dizziness, problems sleeping, scared feelings, vivid dreams, sad feelings that can make you stop doing your usual duties, and vision problems. Mefloquine treatment can also cause convulsions, and mental problems but that is rare. If we give you mefloquine, we will ask you to come back after seven days to check if the malaria has gone.

Like iron tablets, folic acid will help to make enough blood in the body. If you do not take folic acid for a period of two weeks, we believe this will not harm you or your baby, as long as you start taking it after the two weeks.

If we find malaria or a low blood level when we take your blood sample, even if you cannot be in the follow-up study we will give you treatment free of charge for this. If you are infected with the HIV virus you will be able to talk as often as you like with the study HIV worker or nurse. This worker can tell you where to go for medical care if you get sick and how to keep from passing the virus onto others. They can also talk with you about how best to tell others that you have the virus if you decide to do so, and where else you can go in Kisumu to learn more about HIV and to get support.

If you are in the follow-up study, we will give you a treated bed net to protect yourself at night. Because you are getting a baby, and it is important for the baby’s health to be clean and dressed. We will make sure at the end of the follow-up study that you get clothes for this, and Vaseline for the skin. The National Counsel of Churches in Kenya has been giving us items for this purpose.

It is up to you to choose whether you want to be in the study or not. You can join the study but then have questions about the study. Once you join the study, if you decide you do not want to stay in the study, you may leave the study at any time. For questions or any injury that you think may be related to the research, you can contact Dr. Annemieke van Eijk in the New Nyanza Provincial Hospital, telephone 23200, or post address: KEMRI/CDC , P.O. Box 1578 Kisumu. If you have any questions about your rights as a research subject, you may contact Dr. JM Vulule, telephone 22924, Director of KEMRI / Kisumu, PO Box 1578 Kisumu. If you decide not to be screened for the study or to leave the study, you and your child will still receive the best possible care here at the hospital.

Thank you very much for your time. Would you like to join the study?

**The above study has been explained to me and I agree to join.**

1. I have been told about the risks of side effects of SP and the risks and benefits of using folic acid tablets together with SP.
2. I have been told that I cannot continue the study if I get ill and need treatment with a drug that could affect the action of SP or folic acid during the study time.
3. I have been told that during the study, money for transport to the hospital will be given to me.
4. I have been told that it is up to me if I want to join the study and that I can leave the study any time I want. This will not affect the care given to me in this hospital.
5. I have been told that the study doctor may occasionally retrieve my HIV-status if this is important for my medical treatment.
6. I agree to a home visit by the study staff to find out if I still want to stay in the study if I was not able to come to the study clinic.

Participant's statement (signature or thumb print required on appropriate line)

The above has been explained to me:

I agree to join

Participant's name: Date: / /

Witnesses’ statement:

The above consent was explained and the woman agreed to join the study:

Witness

Father’s statement (signature or thumb print required on appropriate line):

The above has been explained to me:

I agree that the baby’s mother joins the study

Father’s name: Date: / /

Witness’ statement:

The above consent was explained and the man agreed for the baby’s mother to join the study:

Witness

Pregnant woman to check if applicable:

____ The father’s consent does not appear because he is not reasonably available, or where the father is, or who he is, is not known for sure, or this pregnancy was the result of a rape.

Participant's name: Date: / /

Witness’ statement:

The woman indicated that the father’s consent could not be obtained:

Witness

Annex 4:

**SCREENING FORM SP-FOLIC ACID IN PREGNANT WOMEN STUDY (SPF-FORM 1a)**

***KEMRI/CDC***

**1.** Code SP [_].[_][_][_][_]

**2.** Date [_][_]\[_][_]\[_][_][_][_]

Interviewer ____________________

**GENERAL INFORMATION**

**3.** How old were you at your last birthday [_][_], if unknown, indicate “99"

Do you know your date of birth? [_] 1=yes 2=no

If yes, birth date [_][_]\[_][_]\[_][_][_][_]

If no, month and year of birth known? [_] 1=yes 2=no

If yes, month and year of birth [_][_]\[_][_][_][_]

If no, year of birth known? [_] 1=yes 2=no

If yes, year of birth [_][_][_][_]

**4.** Place of living: Location code [_][_][_] Sublocation code [_][_][_]

**For coding see “place of living coding sheet”**

Have you within the last month stayed outside of Kisumu for at least one night?

[_] 1=yes 2=no

If yes, where? ________________________________________________

[_] 1= malarious area (rural area) 2=non-malarious area

9=unknown

**5.** Have you ever attended school? [_] 1=yes 2=no 9=don’t know

How many years of schooling did you complete? [_] [_]

What is the highest level of school you attended? [_] 1=Primary school

2=Secondary school

3=College/University (tertiary institutions)

Do you read the newspaper once a week? [_] 1=yes 2=no 9=don’t know

Do you listen to the radio once a week? [_] 1=yes 2=no 9=don’t know

What is your religion? [_] 1=Catholic

2=Protestant/other Christian

3=Muslim

4=no religion

5=Other

If other, specify__________________

What is your ethnic group/tribe? [_] 1=Luo

2=Luhya

3=Other...

If other (3), specify______________

**6.** What type of work do you do? [_]

1=unemployed or looking for work

2=regularly employed full time

3=employed seasonally or on day-to-day basis

4=self-employed (business woman etc)

5=student

6=disabled

7=sick

8=other

If other (8), specify_______________

**7.** Are you now married or living with a man? [_] 1=yes 2=no 9=don’t know

Have you before this partner or ever before been married or lived with a man?

[_] 1=yes 2=no

If yes, what happened with the previous partner? [_] 1=widowed

2=divorced

3=no longer living together

4=other

If other (4), specify_______________

**8.** What is the source of drinking water for members of your household? [_]

1=piped water into house/compound/plot

2=piped water from public tap

3=well water

4=surface water river

5=surface water lake

6=rainwater

7= other

If other (7), specify_____________________

Does your household have Electricity [_] 1=yes 2=no 9=don’t know

A radio [_] 1=yes 2=no 9=don’t know

A television [_] 1=yes 2=no 9=don’t know

A refrigerator [_] 1=yes 2=no 9=don’t know

Could you describe the main material of the roof of your home? [_]

1=grass/thatch

2=iron

3=tiles

What are the walls of your house made of? [_]

1=mud

2=cement covered mud

3=block/bricks

4=other

If other (4), specify_____________________________

What kind of windows does your house have? [_]

1=none or open

2=covered with wood/reeds

3=covered with glass/screens

What type of toilet does your house have? [_]

1=flush toilet

2=latrine

3=other

If other (3), specify_____________________________

Does any member in your household own: A bicycle [_] 1=yes 2=no 9=don’t know

A motorcycle [_] 1=yes 2=no 9=don’t know

A car [_] 1=yes 2=no 9=don’t know

Cattle/goats or sheep [_] 1=yes 2=no 9=don’t know

Chicken [_] 1=yes 2=no 9=don’t know

Land [_] 1=yes 2=no 9=don’t know

For the last week, how many people have lived in your household (including the pregnant woman)? [_][_] (99 if unknown)

How many of these were children less than 5 years? [_][_]

How much money did you use in the last week for food? [_]

1 <100 Ksh per week

2 100-200 Ksh per week

3 200-500 Ksh per week

4 500-1000 Ksh per week

5 > 1000 Ksh per week

8 Does not like to answer

9 Does not know

Do you grow vegetables in your own garden? [_] 1=yes 2=no 9=don’t know

MEDICAL/OBSTETRICAL HISTORY

**9.** Number of previous children who were born alive [_][_]

Number of previous children who were born dead (still births) [_][_]

Number of previous abortions [_][_]

Total Number of previous pregnancies [_][_]

Number of previous life born children who are still alive [_][_]

When did your last pregnancy end? [_][_]/[_][_]/[_][_][_][_]

If unknown, how long was this ago (in months)

How did your last pregnancy end? [_]

1=live birth

2=still birth

3=abortion or miscarriage

If live birth, is he still alive now? [_] 1=yes 2=no

If dead, how old was the child when he/she died? [_][_][_]

**10.** No of weeks gestation (ANC) according to palpation today [_][_]

Date of last menstrual period known [_] 1=yes 2=no

If yes, date of first day of last menstrual period [_][_]\[_][_]\[_][_][_][_]

Do you feel the movements of the baby [_] 1=yes 2=no

Did you feel like having fever in the last 7 days? [_] 1=yes 2=no

**11.** Have you been treated for malaria in the past 4 weeks? [_] 1=yes 2=no

If yes, what treatment did you receive? **(Write name drug, number of tablets per day, and number of days)**

________________________________________________________________________

Have you used any of the following drugs in the last 4 weeks?

Folic acid tablets [_] 1=yes 2=no 9=don’t know

Septrin (other names for septrin: look up) [_] 1=yes 2=no 9=don’t know

Metakelfin [_] 1=yes 2=no 9=don’t know

Fansidar (other names for SP: look up) [_] 1=yes 2=no 9=don’t know

Have you ever had an adverse reaction to a drug, like skin rash, jaundice or vomiting?

[_] 1=yes 2=no 9=don’t know

If yes, what drug?

If yes, what reaction? __________________________________________

Are you currently using any medicine? [_] 1=yes 2=no

If yes: what medicine?

Are you known with any illness on the moment? [_] 1=yes 2=no 9=don’t know

If yes, what illness? ________________________________________________

Are you on the moment

Using treatment for tuberculosis? [_] 1=yes 2=no 9=don’t know

Using treatment for sickle cell disease [_] 1=yes 2=no 9=don’t know

During this pregnancy, have you been admitted in hospital?

[_] 1=yes 2=no 9=don’t know

Date of hospitalization: [_][_]/[_][_]/[_][_][_][_]

If yes, for what problem? ____________________________________________________

**12.** Copy from the ANC card:

Date of the first prenatal visit [_][_]/[_][_]/[_][_][_][_]

Gestational age at first visit (weeks) [_][_] (99 if unknown)

Total number of prenatal visits so far (include this visit) [_][_] (99 if unknown)

Examination:

**13.** Axillary temperature [_][_].[_]^o^C

**14.** Weight [_][_].[_] kg (without shoes)

**15.** Height [_][_][_] cm (without shoes)

**16.**  Conclusion: [_]

1) Candidate: Randomized into group [_] 1 = group A

2 = group B

3 = group C

2) Excluded: reason:

**17.** Checklist for enrolled women:

Folic acid tablets explained and supervised: [_]

SP tablets supervised: [_]

Iron tablets explained and given: [_]

Follow up appointment and card made [_]

Address form made [_]

Home drive arranged [_]

Staff ___________signature, date

**LABORATORY SCREENING FORM SP-FOLIC ACID IN PREGNANT WOMEN STUDY**

**(SPF-FORM 1b)**

***KEMRI/CDC***

**1.** Code SP [_].[_][_][_][_]

**2.** Date sample taken: [_][_]/[_][_]/[_][_][_][_]

**3.** Urine: Taken [_] 1=yes 2=no

Sulfa test [_] 1=positive 2=negative 9=unknown

**4.** Hb [_][_].[_] g/dl

**5.** Blood slide for malaria [_] 1=positive 2=negative 9=not done

If positive for malaria: count [_][_][_][_][_] / 300 WBC

Species: [_]

1 = plasmodium falciparum

2 = plasmodium falciparum and malariae

3 = plasmodium malariae

4 = plasmodium ovale

5=other, specify__________________________________

**6.** Unigold [_] 1=positive 2=negative 3=indeterminate 9=not done

1. Determine [_] 1=positive 2=negative 3=indeterminate 9=not done

**8.** Capillus [_] 1=positive 2=negative 3=indeterminate 9=not done

**9.** Sickle cell test [_] 1=sickle cell disease 2=sickle cell carrier

3= indeterminate status 4=No sickle cell carrier or disease

9=not done

**10.** Blood for folic acid taken [_] 1=yes 2=no

Place of storage in Kisian: _________________________________________________

**FOLLOW UP FORM** **SP-FOLIC ACID IN PREGNANT WOMEN STUDY (SPF-FORM 2)**

***KEMRI/CDC***

**1.** Code SP [_].[_][_][_][_]

1. Date [_][_]/[_][_]/[_][_][_][_]

Interviewer ____________________

1. Type of visit: [_] 1=Scheduled visit (day 2, 3, 7, 14, 21 and 28)

2=Visit to make up for a missed scheduled visit

3=Unscheduled visit (any other day)

Number of days past treatment with SP [_][_]

**4.** Did you use folic acid/placebo every day for the days between this visit and your last visit?

[_] 1 = yes 2=no

If no, how many days did you not take the tablets [_][_]

Did you bring your tablets folic acid (or placebo)? [_] 1=yes 2=no

If yes, number of tablets in the container [_][_]

**5.** Complaints: [_] 1=yes 2=no

If yes, specify

Axillary temperature: [_][_].[_] ^o^ C

**6.** Side-effects:

complaints of itching [_] 1=yes 2=no 9 = unknown

Complaints of rash [_] 1=yes 2=no 9 = unknown

Complaints of blisters [_] 1=yes 2=no 9 = unknown

Anything else [_] 1=yes 2=no 9 = unknown

If yes, specify

**If yes, on any of the above complaints: refer to the CO or study physician**

1. Did you use any medicine between your last and the present visit? [_] 1=yes 2=no

If yes, what medicine did you use, and for how long?

_________________________________________________________________________

**8.** Blood smear: Result: [_] 1=positive 2=negative 9=not done

If positive for malaria: count [_][_][_][_][_] / 300 WBC

Species: [_]

1 = plasmodium falciparum

2 = plasmodium falciparum and malariae

3 = plasmodium malariae

4 = plasmodium ovale

5=other, specify__________________________________

**9.** Blood for folate serum levels taken on day 7: [_] 1=yes 2=no 8 = not applicable

If yes, place of storage in Kisian: ____________________________________________

**10.** Hemoglobin (only on day 14 and 28 or on request): Hb [_][_].[_] g/dl

**11.**  Additional history and examination: (Examining clinical officer or physician ____________)

**12.**  Diagnosis (if complaints): **______________________________________________________**

**13.**  Medication given in study clinic, or other action taken: (if any)

**______________________________________________________________________________**

**14.** Conclusion: [_] 1) Continue, next appointment: [_][_]/[_][_]/[_][_]

2) Excluded: reason: [_]

3) Dropped out: reason:

4) End of study after 28 days

Reasons for exclusion:

1. Use of other sulfa containing drugs or antimalarials when being in the study.

2. RIII resistance: -A parasite density at Day 3 that is > 25% of the Day 0 parasite density.

3. RII resistance: -Parasites at 7 days and a positive blood smear at day 3 with a parasite density that is < 25% of the Day 0 parasite density.

4. Early RI: -A negative Day 3 blood smear with a positive blood smear on any day between Day 4 and Day 14 or a positive Day 3 blood smear with a parasite density that is < 25% of Day 0, a negative Day 7 blood smear, and a positive blood smear on any day between Day 8 and Day 14.

5. Sensitive or late RI: -A Day 3 parasite density that is <25% of the Day 0 density and negative blood smears on every follow-up examination between Day 7 and Day 14.

6. Other reason, specify_________________________________________________

**In case of clinical or parasitologic failure: treat with Quinine 600 mg TDS for 7 days, and make a new appointment after 7 days.**

**For women severely ill, arrange admission in the Provincial Hospital**

**Annex 5: Definition of treatment failure SP-Folate study**

**Early treatment failure**:

- Development of **danger signs** or severe malaria on day 1, day 2, or day 3, in the presence of parasitemia.

**Danger signs** or signs of severe malaria in adults:

Clinical:

- - - Prostration (can’t sit up)
    - Impaired consciousness (not responding to voice or pain stimulus)
    - Respiratory distress (difficulty in breathing and fast breathing
    - Multiple convulsions (spasms of the muscles with jerky movements)
    - Circulatory collapse (fainting)
    - Pulmonary oedema (radiological)
    - Abnormal bleeding (bleeding from nose, ears, mouth or any other body opening)
    - Jaundice (yellow eyes)
    - Haemoglobinuria (blood in urine)

Laboratory:

- - - Severe anemia (Hb < 7 g/dl)
    - Hypoglycemia
    - Acidosis
    - Hyperlactataemia
    - Hyperparasitemia
    - Renal impairment
- Parasitemia on day 2 higher than day 0 count, irrespective of axillary temperature
- Parasitemia on day 3 with an axillary temperature ≥ 37.5
- Parasitemia on day 3 ≥ 25% of count on day 0

**Late clinical failure**:

- Development of danger signs of severe malaria after day 3 in the presence of parasitemia without previously meeting any of the criteria of *Early Treatment Failure*
- Presence of parasitemia and an axillary temperature ≥ 37.5 ºC on any day from day 4 to 28 without previously meeting any of the criteria of *Early Treatment Failure*

**Late parasitological failure**:

- Presence of parasitemia on any day from day 7 to 28 and an axillary temperature of < 37.5 ºC without previously meeting any of the criteria of *Early Treatment Failure* or *Late Clinical Failure*

**Interpretation of definition of treatment failure for the SP-folate staff (to be included in each participant file):**

**Visit result table**

| **Day** | **Day0** | **2** | **3** | **7** | **14** | **21** | **28** |  |
| --- | --- | --- | --- | --- | --- | --- | --- | --- |
| Blood smear result * |  |  |  |  |  |  |  |  |
| Axillary temperature** |  |  |  |  |  |  |  |  |
| Danger signs |  | Yes / No | Yes / No | Yes / No | Yes / No | Yes / No | Yes / No | Yes / No |
| Outcome |  | Continue / Stop | Continue / Stop | Continue  / Stop | Continue  / Stop | Continue  / Stop | Continue  / Stop | Continue  / Stop |

*Parasite density per 300 WBC **In degrees Celsius

**Danger signs** or signs of severe malaria in adults:

**Clinical**: Prostration (can’t sit up), Impaired consciousness (not responding to voice or pain stimulus), Respiratory distress (difficulty in breathing and fast breathing, Multiple convulsions (spasms of the muscles with jerky movements), Circulatory collapse (fainting), Pulmonary oedema (radiological), Abnormal bleeding (bleeding from nose, ears, mouth or any other body opening), Jaundice (yellow eyes), Haemoglobinuria (blood in urine)

**Laboratory**: Severe anemia (Hb < 7 g/dl), Hypoglycemia, Acidosis, Hyperlactataemia, Hyperparasitemia (parasite count ≥ 3750/300 WBC), Renal impairment

| **Day** | **Findings** | **What to do:** |
| --- | --- | --- |
| **1** | Parasitemia of 0 | Continue |
| **1** | Parasitemia < day 0, and no danger signs of severe malaria | Continue |
| **1** | Parasitemia (any density) and axillary temperature ≥ 37.5 ºC | Continue, give Paracetamol |
| **1** | **Danger signs in the presence of parasitemia** | **Treatment failure, exclude** |
| **2** | Parasitemia of 0 | Continue |
| **2** | Parasitemia < day 0, and no danger signs of severe malaria | Continue |
| **2** | Parasitemia < day 0, and an axillary temperature ≥ 37.5 ºC | Continue, give Paracetamol |
| **2** | **Parasitemia > day 0** | **Treatment failure, exclude** |
| **2** | **Danger signs in the presence of parasitemia** | **Treatment failure, exclude** |
| **3** | Parasitemia of 0 | Continue |
| **3** | Parasitemia < 25% of day 0, and no danger signs, and an axillary temperature < 37.5 ºC | Continue |
| **3** | **Parasitemia < 25% of day 0, and an axillary temperature ≥ 37.5 ºC** | **Treatment failure, exclude** |
| **3** | **Parasitemia ≥ 25% of day 0 count** | **Treatment failure, exclude** |
| **3** | **Danger signs in the presence of parasitemia** | **Treatment failure, exclude** |
| **4-6** | Parasitemia of 0 | Continue |
| **4-6** | Parasitemia and no danger signs and an axillary temperature < 37.5 ºC | Continue |
| **4-6** | **Parasitemia > 0 and an axillary temperature ≥ 37.5 ºC** | **Treatment failure, exclude** |
| **4-6** | **Danger signs in the presence of parasitemia** | **Treatment failure, exclude** |
| **7-28** | Parasitemia of 0 | Continue |
| **7-28** | **Any parasitemia**  **(either or not danger signs or an axillary temperature ≥ 37.5 ºC)** | **Treatment failure, exclude** |

**If treatment failure, treat with Quinine.** Make a check appointment for after one week.
